# Venetoclax for elderly patients with acute myeloid leukaemia unfit for intensive chemotherapy: Prospects, resistance mechanisms and management strategies

**DOI:** 10.1002/ctm2.70797

**Published:** 2026-08-28

**Authors:** Jinlin Zhang, Jile Liu, Mohan Zhao, Xiaoyu Zhang, Linlin Liu, Mengnan Li, Changze Cao, Rui Zhang, Mingfeng Zhao

**Affiliations:** ^1^ First Central Hospital of Tianjin Medical University Tianjin Medical University Tianjin China; ^2^ The First Clinical Medical College Shandong Medical and Pharmaceutical University Shandong China; ^3^ School of Medicine Nankai University Tianjin China; ^4^ Department of Hematology Tianjin First Central Hospital Tianjin Thrombosis and Hemostasis Institute Tianjin China

**Keywords:** AML, BCL2 inhibition, elderly patients, resistance mechanisms, therapeutic strategies, venetoclax

## Abstract

To date, the treatment of acute myeloid leukaemia (AML) remains a major challenge in hematologic malignancies, particularly among elderly patients who are unfit for intensive chemotherapy. Venetoclax, a BCL‐2 inhibitor, in combination with azacitidine, has significantly improved the prognosis and survival of this patient population. However, approximately 30% of patients do not respond to venetoclax‐based combination regimens, and most initial responders eventually experience relapse. Therefore, resistance to venetoclax has become a critical bottleneck limiting its long‐term clinical benefits. A thorough understanding of the underlying resistance mechanisms and the development of novel strategies to overcome resistance are central to improving survival outcomes in elderly AML patients. The resistance mechanisms of venetoclax are complex and multifaceted, including genetic drivers such as TP53 mutations, FLT3‐ITD, and NRAS/KRAS mutations, as well as the upregulation of anti‐apoptotic proteins (e.g., MCL‐1, BCL‐xL), metabolic reprogramming, and altered cellular differentiation states. In response to these factors, emerging therapeutic strategies are rapidly evolving toward precision‐based and combination approaches, including combinations with targeted agents (e.g., IDH2 inhibitors, FLT3 inhibitors), MCL‐1 inhibitors, optimization of current dosing regimens (e.g., DEC3‐VEN), and the development of novel modalities (e.g., antibody‐drug conjugates, CAR‐T cell therapy). Focusing on the disease characteristics of elderly AML, this narrative review summarizes the clinical efficacy of venetoclax, critically examines the multifaceted mechanisms of venetoclax resistance, and discusses current and emerging therapeutic strategies to overcome this resistance. The goal is to inform clinical practice and guide future translational research.

**Key Points:**

- This review elucidates the current mechanism of action, therapeutic effects, and limitations of venetoclax, focusing on its use and combination regimens in the specific patient population of elderly individuals with AML who are not suitable for intensive therapy, as well as strategies to overcome resistance, and provides future perspectives for addressing venetoclax resistance.

## Graphical abstract

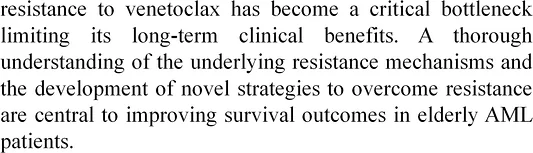

## 1 CURRENT LANDSCAPE OF DIAGNOSIS AND TREATMENT FOR ELDERLY AML PATIENTS UNFIT FOR INTENSIVE CHEMOTHERAPY

### 1.1 Disease characteristics of elderly AML

Acute myeloid leukaemia (AML) is a heterogeneous hematologic malignancy associated with a generally poor clinical prognosis. Approximately 70% of patients diagnosed with AML are aged 65 years or older.^1^ Owing to the frequent coexistence of multiple comorbidities (e.g., cardiovascular disease, diabetes mellitus, chronic kidney disease) and diminished organ function reserve in the elderly population, their tolerance to therapeutic interventions is significantly lower than that of younger patients.^2^, ^3^ According to the relevant literature, elderly patients with AML who exhibit disease characteristics unrelated to leukaemia, such as an age >75 years, congestive heart failure or documented cardiomyopathy with an ejection fraction (EF) ≤50%, and an Eastern Cooperative Oncology Group (ECOG) performance status ≥3, have been defined as a population unfit for intensive chemotherapy.^4^ The 2025 guidelines issued by the American Society of Hematology (ASH) emphasize that treatment decisions for elderly AML patients should integrate multidimensional factors, including chronological age, comorbidity index, performance status (ECOG), cognitive function and social support.^5^

### 1.2 Efficacy of venetoclax‐based combination regimens

The mutational profile of elderly AML patients exhibits distinct age‐related characteristics. The incidence of TP53 mutations increases markedly with advancing age, reaching 15%–20% in patients aged over 75 years, and is frequently accompanied by complex karyotypic abnormalities.^6^, ^7^, ^8^ Mutations in the RAS signalling pathway (NRAS/KRAS) are more prevalent in older patients and mediate resistance to various therapeutic modalities through activation of the mitogen‐activated protein kinase (MAPK) signalling pathway.^6^ Furthermore, mutations in clonal haematopoiesis of indeterminate potential (CHIP)‐related genes (e.g., TET2, DNMT3A, ASXL1), which are closely associated with the ageing process, are highly prevalent in elderly AML. These genes not only participate in the pathogenesis of leukaemogenesis but also modulate mitochondrial metabolism and epigenetic regulation, thereby further increasing disease heterogeneity.^9^ The bone marrow microenvironment in elderly AML patients displays significant age‐associated alterations, primarily characterized by reduced self‐renewal and differentiation capacity of hematopoietic stem cells (HSCs), an increased proportion of marrow adipocytes, functional impairment of stromal cells, and dysregulated secretion of inflammatory cytokines (e.g., IL‐6, TNF‐α).^10^, ^11^ The ageing process is accompanied by declining mitochondrial function, reduced oxidative phosphorylation (OXPHOS) efficiency and elevated oxidative stress. Given the poor baseline metabolic reserve in elderly patients, they are more susceptible to developing therapeutic resistance through metabolic remodelling (e.g., enhanced glycolysis or fatty acid oxidation) under therapeutic pressure.^2^, ^3^ Meanwhile, elderly patients generally exhibit immunosenescence, characterized by reduced T‐cell diversity, impaired effector function, an increased proportion of regulatory T cells (Tregs) and diminished natural killer (NK) cell cytotoxicity.^11^ In addition, the elderly body exists in a state of chronic low‐grade inflammation (“inflamm‐ageing”), wherein the inflammatory microenvironment can activate survival signalling pathways within leukaemia cells and promote the secretion of protective cytokines by bone marrow stromal cells, collectively mediating immune evasion and treatment resistance.^10^, ^11^

Although conventional intensive chemotherapy regimens have yielded certain advances in the treatment of AML, a substantial proportion of elderly patients are unable to tolerate such intensive therapeutic approaches. Prior to the advent of novel agents, treatment options for this patient subgroup were largely limited to supportive care.^12^ The introduction of venetoclax, a selective BCL‐2 inhibitor, has dramatically transformed the clinical outcomes for this AML subgroup. In particular, the combination of venetoclax with hypomethylating agents has provided a more effective therapeutic option for elderly AML patients unfit for intensive chemotherapy.^1^, ^13^ The current standard regimen of venetoclax plus azacitidine (azacitidine 75 mg/m^2^ administered subcutaneously on Days 1–7, and venetoclax 400 mg once daily for 28 consecutive days) was validated in the pivotal phase III VIALE‐A trial involving elderly AML patients ineligible for intensive chemotherapy. This combination regimen achieved an overall response rate of approximately 65%, a 2‐year overall survival (OS) rate of 37.5%, and significantly prolonged the median OS from 9.6 months with azacitidine monotherapy to 14.7 months with the combination.^13^, ^14^ The latest 3‐year follow‐up data from this study further support the efficacy of the combination regimen, demonstrating a 3‐year OS rate of 25% in the venetoclax‐azacitidine group compared with only 10% in the azacitidine monotherapy group.^15^ Currently, given its favourable efficacy and relatively low treatment intensity, this combination regimen has established itself as the standard first‐line treatment for this patient population.^1^ The 2025 ASH treatment guidelines for elderly AML patients unfit for intensive chemotherapy explicitly recommend hypomethylating agent plus venetoclax regimens for older AML patients unsuitable for conventional induction therapy, and suggest continuation of this regimen until disease progression or unacceptable toxicity, rather than discontinuation after a fixed number of treatment cycles.^5^

### 1.3 The limitations of venetoclax‐based combination regimens

The widespread clinical application of venetoclax‐based combination regimens has gradually delineated their safety profile. Based on data derived from the VIALE‐A study, the most common grade ≥3 adverse events observed in the venetoclax‐azacitidine (VEN‐AZA) group were thrombocytopenia (45%), febrile neutropenia (42%), and neutropenia (42%). Among serious adverse events, pneumonia (22%), sepsis (19%), and febrile neutropenia (30%) represented the most prominent infectious complications. The incidence of bleeding events was 6%. The safety profile was generally consistent across age subgroups (75–79 years, 80–84 years, and ≥85 years), with no novel safety signals identified.^12^, ^13^, ^14^, ^15^ Furthermore, tumour lysis syndrome (TLS) is overall rare in AML (only three cases were reported in the VIALE‐A trial); however, given its potential severity, hydration and biochemical monitoring are still recommended at the initiation of treatment. Real‐world data further highlight that the infectious risk associated with cytopenias constitutes a core challenge in clinical management. In a real‐world analysis involving 106 patients, the incidence of febrile neutropenia and new‐onset infections during the first treatment cycle was 27% and 25%, respectively; these complications were significantly associated with inferior survival outcomes (median OS 4.9 months in patients experiencing complications vs. 11.6 months in those without, *p* = 0.03).^16^ Notably, due to the potential for higher venetoclax exposure levels in Asian populations, several real‐world studies from China have suggested that, under close clinical monitoring, shortening the duration of venetoclax administration or reducing its dose may be considered to optimize the toxicity‐efficacy balance. Overall, the toxicity spectrum of the VEN‐HMA regimen is dominated by hematologic toxicities and secondary infections. Through individualized dose adjustments (e.g., shortening the 28‐day administration period to 14 or 21 days), prophylactic anti‐infective measures (e.g., fluoroquinolone prophylaxis), and therapeutic drug monitoring, the adverse effects of this combination regimen can be effectively managed.^17^, ^18^

Currently, clear clinical guidance has emerged regarding the optimization of treatment duration and dosing of the VEN‐HMA regimen in AML. Numerous small‐scale retrospective studies have successfully demonstrated that reducing the venetoclax dose or shortening the treatment duration can achieve efficacy comparable to that reported in the VIALE‐A study.^18^ A prospective study by Okta et al. provided high‐level evidence supporting the use of reduced‐dose or short‐duration venetoclax regimens in unfit AML patients. Compared with the standard 28‐day regimen, both the 14‐day standard‐dose regimen (venetoclax 400 mg for 14 days) and the 21‐day reduced‐dose regimen (venetoclax 100 mg for 21 days) resulted in higher response rates, improved overall survival and reduced toxicity.^19^ The VENAZA‐5S trial, presented at the 2025 ASH Annual Meeting (azacitidine 75 mg/m^2^ administered subcutaneously on Days 1–5 of each 28‐day cycle, and venetoclax 400 mg orally once daily on Days 1–28), evaluated the efficacy and safety of shortening the azacitidine administration period from 7 to 5 days. The results demonstrated that this combination regimen achieved favourable efficacy and tolerability (complete cytogenetic response (CCyR) 53.3%, overall response rate (ORR) 60%; median follow‐up of 11.5 months, OS 11.1 months). Grade ≥3 haematologic adverse events (i.e., neutropenia, anaemia, or thrombocytopenia) occurred in 30 patients (67%), and grade ≥3 infections in 17 patients (38%), thereby providing a new therapeutic option for patients unable to tolerate the standard 7‐day azacitidine regimen.^20^ Additionally, another study presented at the 2025 ASH Annual Meeting showed that, in newly diagnosed unfit AML patients, a regimen of low‐dose venetoclax (100 mg/days on Days 1–21) plus standard‐dose hypomethylating agent (azacitidine 100 mg/day on Days 1–7) combined with itraconazole (100 mg twice daily on Days 1–21) achieved efficacy comparable to that of the 7+3 intensive chemotherapy regimen (59.5% vs. 76.9%, *p* = 0.10), with a more favourable safety profile (febrile neutropenia rate 50% vs. 90.6%, *p* = 0.0001; 60‐day mortality 4.5% vs. 26.8%, *p* = 0.003; median hospital stay 14 days vs. 28 days, *p* = 0.0001). This regimen not only reduces treatment costs but also enables outpatient administration, offering a more feasible first‐line alternative for resource‐limited settings and for elderly or frail patients with poor performance status^21^ (Table 1).

**TABLE 1:** Summary of clinical efficacy and safety of venetoclax‐based regimens in elderly AML patients unfit for intensive chemotherapy.

First Author	Patient Population	Dosing Regimen	Efficacy	Adverse Events	Reference
Courtney D. DiNardo	Newly diagnosed AML, ≥75 years	Venetoclax (400 mg, Days 1–28, ramp‐up in cycle 1) + azacitidine (75 mg/m^2^, Days 1–7, SC/IV), 28‐day cycle	AZA + VEN: median OS 14.7 months vs placebo + AZA 9.6 months; 46% reduction in risk of death	Grade ≥3: thrombocytopaenia 45%, neutropenia 42%, febrile neutropaenia 42%; serious: pneumonia 22%	^12^
Chen Xiuli	Elderly newly diagnosed AML (*n *= 17), median age 70 years	Same as above	CRc 64.7%, ORR 88.2%, MRD negativity 52.9%; median PFS 6.5 months, median OS 12.0 months	Hematologic AEs 100% (94.1% grade ≥3); infection 88.2% (pulmonary 82.4%)	^22^
Kristjan Paulson	Newly diagnosed unfit AML (*n* = 185), median age 76 years	Same as above	CR/CRi 66.7%; median time to response 71 days; TP53‐mutant subgroup CR/CRi 65.4%	Grade ≥3 TEAEs 71.9%, grade ≥3 hematologic TEAEs 66.5%; neutropenia 45.4%	^23^
Pollyea DA	Newly diagnosed unfit elderly AML, median age 74 years	VEN + AZA (same as above) or VEN + DEC (decitabine 20 mg/m^2^, Days 1–5, IV)	DEC+VEN: CR/CRi 74%, median OS 16.2 months; AZA + VEN: CR/CRi 71%, median OS 16.4 months	Febrile neutropenia: DEC + VEN 65%, AZA + VEN 39%	^24^
Daehun Kwag	Newly diagnosed elderly AML (*n* = 74), median age 71 years	VEN + HMA (standard dose, details not specified)	Combination group: median OS 13.4 months vs DEC monotherapy 8.3 months (*p* = .01); ORR 70.3% vs. 24.3%; CR/CRi 67.6%	No significant difference in safety between groups (grades not detailed)	^25^
Maiti A	Newly diagnosed elderly AML (*n* = 170), median age 72 years	VEN + HMA (standard dose)	VEN + HMA: CR/CRi 81% vs. intensive chemotherapy 52%; OS 18.1 months vs. 13.4 months	30‐day mortality 1% vs. 24%; lower infectious complications	^26^
Imanaka R	Newly diagnosed unfit AML (*n* = 120), median age 77 years, 52% s‐AML	VEN + HMA (standard dose)	CR/CRi 56.7%; median OS 14.8 months	Not reported in detail	^27^

Abbreviations: AZA, azacitidine; CR, complete remission; CRc, composite CR (including CR/CRi); CRi, CR with incomplete hematologic recovery; DEC, decitabine; HMA, hypomethylating agent; mOS, median overall survival; ORR, overall response rate; PFS, progression‐free survival; R/R, relapsed/refractory; unfit, unfit for intensive chemotherapy; VEN, venetoclax; VA, venetoclax + azacitidine.

The current venetoclax plus hypomethylating agent (VEN‐HMA) regimen is confronted with two major clinical challenges. On one hand, approximately 30% of patients exhibit primary resistance to this therapeutic regimen.^28^ On the other hand, even among initial responders, disease relapse frequently occurs following a median remission duration of approximately 18 months, which is indicative of the development of acquired resistance. Moreover, for elderly patients with relapsed/refractory (R/R) AML who experience treatment failure with the VEN‐HMA regimen, the clinical prognosis is extremely poor, characterized by a significantly shortened median overall survival.^29^, ^30^, ^31^ Therefore, in‐depth investigation into the molecular mechanisms underlying venetoclax resistance and the exploration of targeted, effective therapeutic strategies directed against these mechanisms are of paramount importance for improving clinical outcomes in this vulnerable patient population.

## 2 VENETOCLAX MECHANISM OF ACTION

### 2.1 Core regulators of the intrinsic apoptotic pathway: The BCL‐2 protein family

To gain an in‐depth understanding of the therapeutic mechanisms of venetoclax and the origins of drug resistance, it is first necessary to delineate the central role of the BCL‐2 protein family in the regulation of apoptosis and its aberrant expression in AML. Defects in apoptotic signalling represent a well‐recognized hallmark of tumorigenesis, and the BCL‐2 protein family serves as the central regulatory hub of the mitochondrial intrinsic apoptotic pathway.^26^, ^27^ This family is primarily categorized into three functionally distinct subfamilies: (1) Anti‐apoptotic proteins (including BCL‐2, BCL‐xL, MCL‐1, BCL‐w, and A1), which sustain cellular survival by directly inhibiting the activity of pro‐apoptotic proteins^32^, ^33^; (2) pro‐apoptotic BH3‐only proteins, further subdivided into apoptosis‐sensitizing molecules (e.g., BAD, BIK, HRK, NOXA) and apoptosis‐activating molecules (e.g., BIM, BID, PUMA), which can either directly activate effector proteins or neutralize the anti‐apoptotic functions of BCL‐2 family members^34^, ^35^, ^36^; (3) effector proteins (BAX and BAK) disrupt mitochondrial membrane integrity and induce mitochondrial outer membrane permeabilization (MOMP), a critical, irreversible step in the execution of apoptotic cell death.^37^ Under physiological conditions, anti‐apoptotic proteins such as BCL‐2 prevent the activation of the pro‐apoptotic proteins BAX and BAK via their BH3 domains, thereby preserving mitochondrial membrane integrity and maintaining cellular homeostasis.^32^, ^33^ Upon apoptotic stimulation, BH3‐only proteins are activated and competitively bind to anti‐apoptotic proteins, thereby releasing sequestered pro‐apoptotic proteins and subsequently triggering the activation of BAX and BAK.^34^, ^35^ Activated BAX and BAK undergo conformational changes and oligomerize on the outer mitochondrial membrane to form transmembrane pores, leading to MOMP and the release of apoptogenic factors (e.g., cytochrome c) into the cytoplasmic compartment. This event initiates the caspase activation cascade, ultimately culminating in apoptotic cell death.^32^, ^38^

**FIGURE 1:**
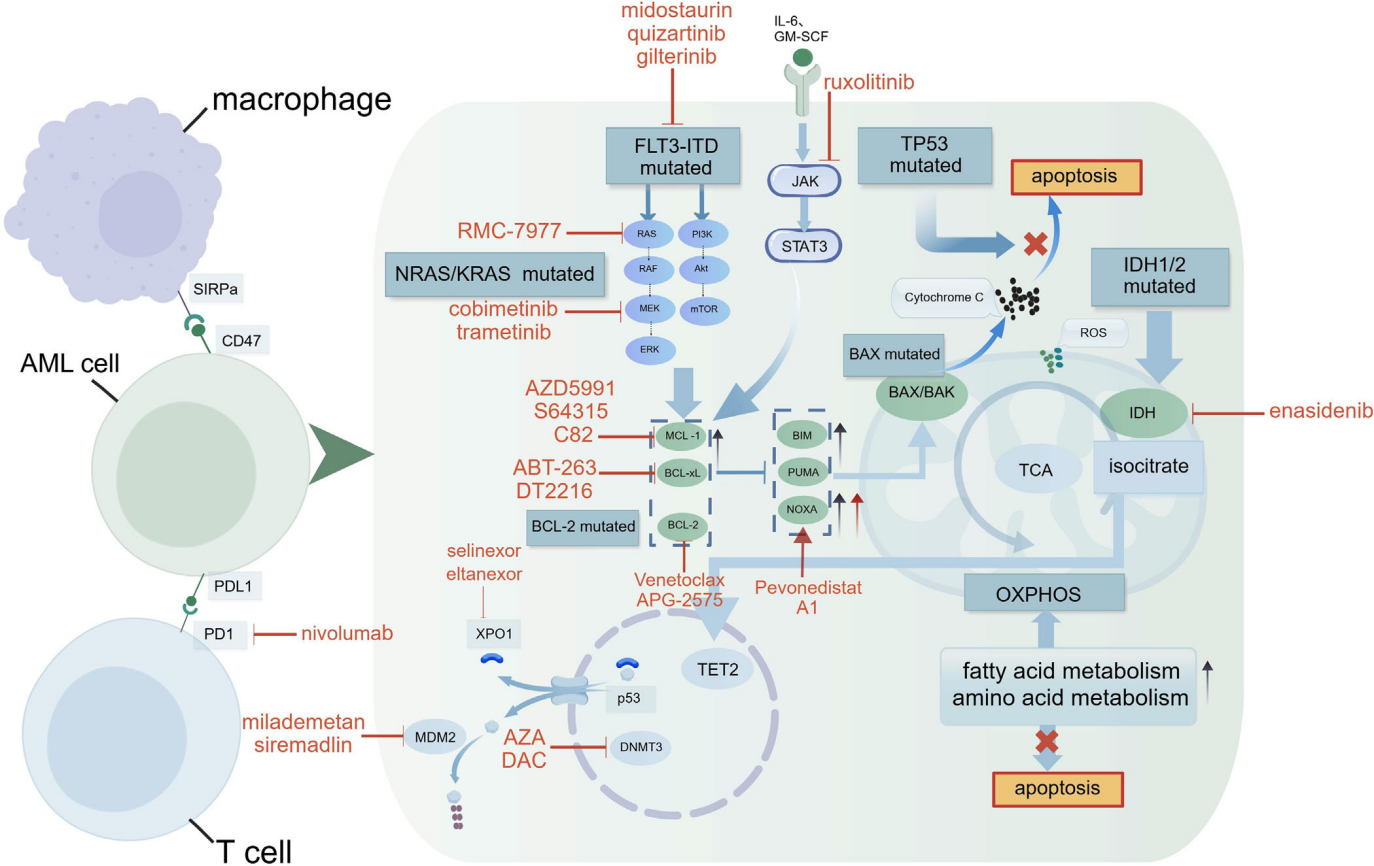
Mechanisms of resistance to venetoclax therapy and therapeutic strategies in AML. AZA, 5‐azacitidine; DAC, decitabine; DNMT3A, DNA methyltransferase 3A; FLT3, FMS‐like tyrosine kinase 3; IDH, isocitrate dehydrogenase; ITD, internal tandem duplication; MDM2, E3 ubiquitin–protein ligase; MYC and p53, transcription factors; OXPHOS, oxidative phosphorylation; ROS, reactive oxygen species; TET2, tet methylcytosine dioxygenase 2; TCA, tricarboxylic acid cycle; XPO1, exportin 1.

In AML cells, upregulation of anti‐apoptotic proteins, transcriptional suppression of the BH3‐only proteins BIM and NOXA, and aberrant overexpression of BCL‐2 and MCL‐1 collectively sustain leukaemic cell survival. Among these alterations, elevated expression of anti‐apoptotic BCL‐2 family members not only supports the survival and persistence of leukaemic stem cells (LSCs) in AML but also correlates closely with chemoresistance phenotypes. Accumulating evidence has demonstrated that BCL‐2 overexpression can be driven by multiple molecular mechanisms, including gene hypomethylation, genomic amplification and chromosomal rearrangement.^39^, ^40^, ^41^ In addition, deletion of miR‐15 and miR‐16 at the 13q14 locus abrogates their post‐transcriptional repressive effects on BCL‐2, further contributing to its aberrant upregulation in AML^42^, ^43^, ^44^ (Figure 1).

### 2.2 Venetoclax triggers the apoptotic program in AML cells

Venetoclax functions as a highly selective BH3 mimetic that binds to the BH3 domain of BCL‐2 with exceptionally high affinity.^45^, ^46^ Via competitive occupancy of the BCL‐2 binding groove, venetoclax replaces endogenous pro‐apoptotic BH3‐only proteins that are constitutively sequestered by BCL‐2. The liberated BH3‐only proteins subsequently activate the downstream effector molecules BAX and BAK, thereby initiating the intrinsic apoptotic cascade in AML cells.^47^, ^48^ In contrast to non‐selective pan‐BCL‐2 BH3 mimetics (e.g., ABT‐737, ABT‐263/navitoclax), the predominant superiority of venetoclax lies in its approximately 1000‐fold binding selectivity for BCL‐2 over other anti‐apoptotic homologs, including BCL‐xL and MCL‐1, which allows specific pharmacological targeting of BCL‐2.^45^, ^46^, ^47^, ^48^, ^49^, ^50^ Furthermore, given that platelet viability relies heavily on BCL‐xL‐mediated pro‐survival signals, the exquisite BCL‐2 selectivity of venetoclax circumvents BCL‐xL‐driven platelet suppression and consequent thrombocytopenia, thereby favourably improving its therapeutic safety profile.^44^, ^45^, ^46^

Furthermore, LSCs exhibit a distinct energy metabolic signature, relying predominantly on mitochondrial OXPHOS for energy generation. The maintenance of this unique metabolic phenotype is dependent on BCL‐2 signalling, rendering LSCs directly vulnerable to venetoclax‐mediated elimination via specific BCL‐2 targeting.^9^, ^51^, ^52^ Of note, conventional chemotherapeutic agents primarily target actively proliferating cells in the cell cycle, while demonstrating limited efficacy against LSCs that persist in a prolonged quiescent (G0) state.^53^, ^54^ In contrast, venetoclax disrupts the energy supply of LSCs through BCL‐2 inhibition, thereby effectively eradicating quiescent LSCs that are refractory to conventional chemotherapy.^52^, ^55^ This unique mechanism of action constitutes a critical molecular basis for the achievement of deep remission in AML following venetoclax treatment.^51^, ^55^

Accumulating recent evidence has demonstrated that, in addition to its direct pro‐apoptotic effects, venetoclax can enhance T‐cell‐mediated anti‐leukaemic immune responses.^56^, ^57^ Specifically, venetoclax augments the effector function of CD8^+^ T cells by inducing the generation of mitochondrial reactive oxygen species (mtROS), increases the proportion of naive CD8^+^ T cells, reduces the abundance of immunosuppressive Tregs, and modulates the expression profile of immune checkpoint molecules. These findings not only deepen the understanding of the multifaceted mechanism of action of venetoclax but also provide novel insights and directions for optimizing clinical combination therapeutic strategies.^56^, ^57^, ^58^, ^59^

### 2.3 Venetoclax and HMAs synergistically exert cytotoxic effects

Hypomethylating agents (HMAs), exemplified by AZA and decitabine (DAC), primarily suppress DNA methyltransferase (DNMT) activity. They reverse aberrant hypermethylation of tumour suppressor genes and restore their physiological expression, thereby restraining clonal expansion of AML blasts and facilitating cell differentiation and maturation.^60^ As monotherapy, venetoclax achieves an overall response rate of merely 19% in AML patients, whereas single‐agent HMA therapy, although generally well tolerated, yields modest response rates ranging from 10% to 50% and a median survival of only 6–10 months. Accordingly, this combinatorial regimen has become the standard first‐line option for elderly or medically unfit AML patients ineligible for intensive chemotherapy.^61^, ^62^, ^63^

1. Apoptosis sensitization: HMAs downregulate the expression of the anti‐apoptotic protein MCL‐1 and upregulate the pro‐apoptotic protein NOXA, thereby lowering the apoptotic threshold of AML cells for venetoclax. Notably, the two agents exhibit synergistic effects even at low drug concentrations.^64^
2. Enhanced oxidative stress: Venetoclax disrupts the Nrf2/Keap1 complex and inhibits the Nrf2 antioxidant pathway, thereby increasing mitochondrial ROS production and potentiating the cytotoxic effects of HMAs.^65^
3. Targeting LSCs: HMAs induce differentiation of LSCs, while venetoclax inhibits OXPHOS in differentiated LSCs, thereby synergistically eliminating quiescent LSCs, which is a mechanism critical for achieving deep remission and preventing relapse in AML.^66^, ^67^, ^68^

Based on the synergistic mechanisms described above, venetoclax combined with HMAs has demonstrated superior efficacy and a manageable safety profile over monotherapy in the phase III VIALE‐A trial.^15^, ^67^ Also, real‐world clinical investigations have further reported an overall response rate ranging from 73.8% to 83.7% with a tolerable safety profile. Moreover, a low‐dose weekly DAC plus VEN regimen has yielded a high composite complete remission (CRc) rate of 86.7% in myeloid neoplasms, offering a promising alternative therapeutic strategy for clinical practice.^69^, ^70^

## 3 MULTIDIMENSIONAL ANALYSIS OF VENETOCLAX RESISTANCE MECHANISMS IN ELDERLY AML PATIENTS INELIGIBLE FOR INTENSIVE CHEMOTHERAPY

The clinical efficacy of the venetoclax‐hypomethylating agent combination regimen has been well validated in clinical practice. Nevertheless, the emergence of venetoclax resistance remains a major bottleneck contributing to treatment failure and disease relapse among elderly AML patients. The mechanisms driving venetoclax resistance in this population are multifactorial and intrinsically complex, and are further compounded by physiological ageing, concurrent comorbidities and impaired treatment tolerance. Such resistance primarily arises from genetic aberrations, apoptotic pathway dysregulation, metabolic reprogramming, and tumour microenvironment remodelling—all of which exhibit more pronounced effects in elderly individuals.

### 3.1 Genetic and non‐genetic mechanisms

Mechanisms of venetoclax resistance are multifaceted, involving genetic mutations, compensatory anti‐apoptotic protein upregulation and differentiation‐state heterogeneity. These mechanisms are particularly pronounced in elderly AML patients, who exhibit high mutational burdens, increased clonal heterogeneity, and a dysregulated bone marrow microenvironment—collectively establishing a biological substrate that favours resistance development.^71^

Genetic aberrations constitute a core driver of venetoclax resistance, encompassing both on‐target mutations and other oncogenic alterations.^71^ Acquired mutations in the BCL2 gene (e.g., G101V, Asp103Tyr) markedly reduce venetoclax binding affinity, blunting its pro‐apoptotic activity and representing a key mechanism of acquired resistance in elderly patients. Mutations in TP53, FLT3‐ITD, and RAS are also strongly correlated with venetoclax resistance.^6^, ^9^, ^71^, ^72^ TP53 mutations account for approximately 5%–10% of newly diagnosed AML cases, with higher prevalence in older adults (8.9% in a cohort with median age 67 years).^63^ Mechanistically, mutant p53 loses canonical transcriptional activity and fails to effectively activate downstream pro‐apoptotic targets such as PUMA and BAX. This impairment constrains MOMP initiation and blocks apoptotic execution; consequently, even with pharmacological BCL‐2 inhibition by venetoclax, efficient MOMP activation cannot be achieved, enabling leukaemic cells to evade mitochondria‐driven apoptosis.^73^, ^74^, ^75^, ^76^ Emerging studies have uncovered novel mechanistic insights into TP53‐mediated resistance. CRISPR screening identified ADSS2 as a critical modulator of venetoclax responsiveness; genetic knockout of ADSS2 resensitizes resistant cells to venetoclax. TP53‐mutant cell lines exhibit significant ADSS2 upregulation via c‐Myc–mediated transcriptional activation, and the ADSS2 inhibitor Cmpd3 effectively reduced leukaemic burden in a TP53‐mutant PDX model.^77^ Concurrently, BH3 profiling analyses reveal that TP53‐mutant AML cells retain intact MOMP upon venetoclax + azacitidine exposure, while downstream caspase‐3/7 activation is selectively impeded. Genome‐wide CRISPR screening has pinpointed Survivin (encoded by BIRC5) as the central effector driving this post‐MOMP apoptotic blockade. In TP53‐mutant cells, disruption of the p53/miR‐34a regulatory axis triggers Survivin overexpression; BIRC5 knockout rescues caspase activation and reverses venetoclax resistance.^78^

FLT3‐ITD and RAS mutations primarily mediate moderate venetoclax resistance by activating downstream MAPK and NF‐κB signalling, which drives upregulation of MCL‐1 and BCL‐xL.^6^, ^9^, ^79^, ^80^ Among these, RAS mutations occur invariably late in the course of AML, upon progression or relapsed/refractory disease, which serve as an important predictor of poor therapeutic response in elderly AML patients. RAS‐mutant leukaemia stem cells are observed frequently in patients with poor responses to treatment with the BCL2 inhibitor venetoclax.^81^ In contrast, NPM1 and IDH1/2 mutations typically correlate with favourable responses to venetoclax‐based regimens.^5^, ^8^ Notably, the favourable response conferred by NPM1 mutations is heavily dependent on the co‐mutation background. Real‐world data demonstrate that median OS of elderly patients with isolated NPM1 mutations can reach 22 months; however, concurrent FLT3‐ITD—particularly in the ‘triple mutation’ context (NPM1/FLT3‐ITD/DNMT3A)—significantly reduces median OS to only 10 months.^82^ Data from the 2025 ASH Annual Meeting further clarify that isolated NPM1 mutations remain a favourable prognostic marker in elderly patients receiving VEN + HMA, whereas co‐occurring RAS mutations (especially NRAS) completely abrogate this favourable effect; FLT3‐ITD co‐mutation does not significantly alter the prognosis associated with isolated NPM1 mutations.^83^ With the widespread clinical application of venetoclax‐based regimens, the traditional ELN risk stratification system has encountered limitations.^7^ A four‐gene prognostic model, centred on TP53, FLT3‐ITD and NRAS/KRAS mutations, has been established and validated in real‐world cohorts.^6^, ^7^, ^9^ This model classifies patients into three benefit tiers: high‐benefit (wild‐type for all four genes, 52%), intermediate‐benefit (FLT3‐ITD or NRAS/KRAS mutations, 25%), and low‐benefit (TP53 mutations, 23%). Median OS showed a significant gradient: 26.5, 12.1 and 5.5 months, respectively (*p* <.001).^7^ The PRISM prognostic scoring system, presented at the 2025 ASH Annual Meeting, systematically developed and validated a novel risk stratification tool specifically for newly diagnosed AML patients receiving low‐intensity therapy (HMAs/LDAC) combined with venetoclax. Integrating large‐scale international cohort data, the final PRISM model includes age, sex, sAML‐AHD, ELN 2022‐defined complex karyotype and other adverse‐risk cytogenetic abnormalities, as well as adverse mutations in KRAS, PTPN11, FLT3‐ITD, JAK2, ASXL1, and TP53. Favourable features include diploid karyotype, RUNX1 or IDH2 mutations, and ResMut‐fav (CEBPA, BCOR, IDH1, SF3B1). Beta coefficients from the final robust Cox model are summed to generate a patient‐specific PRISM score; quartiles define four clinical risk groups. In both training and multiple independent validation cohorts, the PRISM C‐index (0.64–0.66) significantly exceeded that of the four‐gene model (0.58–0.61).^84^, ^85^, ^86^ Refined stratification models based on single‐cell sequencing and dynamic disease monitoring are anticipated to further enhance predictive performance.^6^, ^9^

Beyond genetic alterations, compensatory upregulation of anti‐apoptotic proteins represents a core event underlying venetoclax resistance at the protein level.^87^ Under the selective pressure exerted by venetoclax, leukaemic cells can switch their survival dependence from BCL‐2 to other anti‐apoptotic family members, with MCL‐1 being the most well‐characterized mediator. In venetoclax‐resistant AML cells, MCL‐1 expression is significantly upregulated whereas BCL‐2 is downregulated; BH3 profiling confirms that survival dependency has shifted to MCL‐1.^88^, ^89^, ^90^, ^91^ The aberrant accumulation of MCL‐1 is regulated through multi‐level mechanisms: at the transcriptional level, MYC and STAT3 can transactivate MCL‐1 expression^89^, ^91^; at the post‐translational level, AKT‐mediated phosphorylation at Thr163 inhibits its ubiquitination and proteasomal degradation, while the deubiquitinase USP9X further stabilizes MCL‐1.^92^, ^93^, ^94^ Additionally, BCL‐xL exerts a compensatory role in specific contexts such as microenvironment‐mediated resistance.^95^ Concurrently, downregulation of NOXA and increased sequestration of BIM by MCL‐1 (with reduced binding to BCL‐2) collectively reinforce the anti‐apoptotic phenotype.^88^, ^92^ Notably, MCL‐1 exerts pleiotropic effects beyond its canonical anti‐apoptotic function, including regulation of cellular bioenergetics, carbohydrate metabolism, and cell adhesion in AML cells. Its overexpression is associated not only with venetoclax resistance but also with inferior prognosis in elderly AML patients.^96^, ^97^ In elderly AML patients, the dysregulated bone marrow microenvironment and frequent co‐occurring mutations (e.g., FLT3‐ITD, PTPN11) further augment MCL‐1 and BCL‐xL upregulation. FLT3‐ITD promotes BCL‐xL transcription via STAT5 and maintains MCL‐1 stability via PI3K‐Akt, while PTPN11 activating mutations constitutively activate MAPK signalling to synergistically upregulate both proteins.^79^, ^98^ These interconnected mechanisms account for the higher prevalence of such resistance phenotypes in elderly patients.

The heterogeneity of AML cell differentiation status exerts a significant influence on venetoclax sensitivity, a feature particularly prominent in elderly patients, consistent with their impaired differentiation, aberrant blast proportions and high clonal heterogeneity.^99^ AML cells with monocytic (M_4_, M_5_) or erythroid/megakaryocytic differentiation features exhibit markedly reduced or absent BCL‐2 expression, accompanied by enhanced MCL‐1 dependence and stronger venetoclax resistance.^100^, ^101^, ^102^ The resistance of monocytic AML is rooted in a CEBPB‐driven inflammatory positive feedback loop involving IL‐1β/TNF‐α/NF‐κB signalling. This mechanism not only upregulates MCL‐1 but also establishes a protective microenvironment through inflammatory cytokine secretion, collectively contributing to broad‐spectrum resistance.^103^ The incidence of monocytic AML is significantly higher in elderly patients than in younger counterparts.^100^ Clinical data show that the CR rate in monocytic‐differentiated AML is significantly lower than in non‐monocytic AML (59% vs. 71%), with markedly shorter median OS (7.8 months vs. 13.6 months).^102^ Elderly patients frequently present with impaired differentiation; in some cases, IDH2 mutations lead to aberrant hypermethylation that further blocks differentiation, favouring the survival of resistant clones with monocytic or erythroid/megakaryocytic features.^99^ Moreover, aberrant crosstalk between leukaemic cells and the bone marrow stroma can further enhance the survival of resistant clones by modulating MCL‐1 expression.^101^

### 3.2 Metabolic remodelling and microenvironmental alterations

Adaptive metabolic remodelling of leukaemic cells and aberrations in the bone marrow microenvironment are additional critical mechanisms underlying venetoclax resistance in elderly AML patients. Owing to their poor baseline metabolic reserve, elderly patients are more susceptible to developing a venetoclax‐resistant phenotype through adaptive metabolic remodelling under therapeutic pressure. Specifically, venetoclax‐sensitive AML cells rely on BCL‐2 to sustain mitochondrial OXPHOS for energy generation, whereas venetoclax‐resistant AML cells undergo distinct metabolic reprogramming.^9^, ^104^ Specifically, these resistant cells switch to maintaining mitochondrial function and OXPHOS through MCL‐1 to evade venetoclax‐induced apoptosis^92^; in addition, a subset of resistant cells upregulates the activity of glycolysis‐related enzymes, thereby enhancing glycolytic energy metabolism to support their survival.^105^, ^106^

For elderly AML patients, intrinsic metabolic decline, characterized by impaired mitochondrial function, glucose metabolism dysregulation, and more pronounced bone marrow hypoxia, can induce glycolytic reprogramming via the HIF‐1α signalling pathway.^107^ Concurrently, increased oxidative stress enhances MCL‐1 protein stability, which further facilitates metabolic reprogramming in venetoclax‐resistant cells,^92^, ^108^ rendering this type of resistance more likely to occur in the elderly population. Moreover, LSCs frequently exhibit partial differentiation features and aberrantly elevated OXPHOS activity, which contributes to their significantly reduced sensitivity to venetoclax.^9^, ^109^ Preclinical and clinical studies have demonstrated that LSCs primarily depend on OXPHOS for their survival and self‐renewal; the combination of venetoclax with azacitidine blocks OXPHOS by inhibiting amino acid metabolism, thereby triggering LSC apoptosis.^101^ However, venetoclax‐resistant LSCs can acquire a survival advantage by activating compensatory metabolic pathways, such as fatty acid oxidation (FAO).^9^ Notably, in monocytic AML, LSCs retain high BCL‐2 expression and thus remain sensitive to venetoclax, whereas differentiated non‐LSC populations exhibit MCL‐1‐dependent venetoclax resistance. This observation indicates that the persistent survival and clonal expansion of LSCs are critical drivers of venetoclax resistance and disease relapse in elderly AML patients. Therefore, combinatorial therapeutic strategies targeting LSCs and their unique metabolic characteristics hold considerable promise for overcoming venetoclax resistance and improving clinical outcomes.^9^, ^101^, ^109^

The bone marrow microenvironment constitutes a protective niche for AML blasts, and its dysregulation plays a critical role in driving venetoclax resistance among elderly patients. This process is closely linked to age‐related degeneration of the hematopoietic microenvironment and progressive immune senescence.^9^, ^85^, ^110^ In elderly individuals with AML, the bone marrow microenvironment undergoes profound remodelling, manifested primarily by stromal cell dysfunction, aberrant secretion of cytokines including IL‐6, and compromised immune cell activity; collectively, these alterations impair the host's capacity to clear leukaemic cells effectively.^11^, ^111^ Bone marrow stromal cells shield leukaemic cells from venetoclax‐induced apoptosis via direct cell–cell contact or paracrine secretion of soluble mediators. These factors subsequently activate pro‐survival signalling cascades and upregulate the expression of anti‐apoptotic proteins.^85^, ^112^ The hypoxic and nutrient‐deprived microenvironmental milieu further drives metabolic reprogramming, thereby sustaining the survival of LSCs. Given the inherently higher LSC proportion in elderly AML patients, this microenvironmental feature further exacerbates therapeutic resistance and disease relapse.^9^, ^80^, ^107^ Notably, the chronic low‐grade inflammatory state prevalent in aging populations persistently activates the NF‐κB signalling pathway. This cascade stimulates bone marrow stromal cells to secrete elevated levels of pro‐survival cytokines and enhances the adhesive properties of leukaemic cells, facilitating their stable engraftment within the bone marrow niche. Such interactions establish a reciprocal pro‐resistance circuit between leukaemic cells and the bone marrow microenvironment, further attenuating the therapeutic efficacy of venetoclax.^11^, ^85^

Furthermore, CD47 expressed on the surface of AML cells binds to signal regulatory protein α (SIRPα) on bone marrow macrophages, transmitting a canonical ‘don't eat me’ signal that suppresses macrophage‐mediated phagocytosis of leukaemic cells. During clonal evolution driven by the selective pressure of venetoclax therapy, upregulated CD47 expression acts as a pivotal immune evasion mechanism, enabling resistant leukaemic clones to escape immune surveillance and sustain long‐term persistence.^59^, ^113^

The multifaceted and interconnected nature of venetoclax resistance poses substantial challenges for elderly AML patients. These mechanistic insights, however, have laid the groundwork for rationally designed combination strategies aimed at overcoming resistance, which will be discussed in the following section.

## 4 EMERGING THERAPEUTIC STRATEGIES AND RESEARCH PROGRESS FOR OVERCOMING VENETOCLAX RESISTANCE

In response to the aforementioned resistance mechanisms, extensive preclinical and clinical investigations are actively exploring multi‐level therapeutic interventions. Current research directions focus on several key areas: combination regimens targeting driver mutations or core resistance‐associated proteins, optimization of dosing strategies, remodelling of metabolic pathways or the bone marrow microenvironment, and modulation of anti‐tumour immune responses. These strategies address distinct resistance mechanisms and collectively provide multiple potential approaches to overcome venetoclax resistance in elderly AML patients (Table 2).

**TABLE 2:** Clinical trials of combination strategies to overcome venetoclax resistance in elderly unfit AML patients.

First author	ClinicalTrials.gov Identifier	Drug Regimen	Target/Mechanism	Patient population	Efficacy	Common grade ≥3 adverse events	Survival	Reference
Newly diagnosed elderly AML								
Short NJ	NCT04140487	Gilteritinib + VA	FLT3 inhibitor	FLT3‐mutant AML (*n* = 30); median age 71y	Treatment‐naïve CR/CRi 96%;	Grade 3 or higher infection 53%;febrile neutropaenia 33%	The mOS has not been reached	^117^
Yilmaz	NCT03661307	Quizartinib + DEC +VEN	FLT3 inhibitor	FLT3‐mutant AML (*n* = 10); median age 70 y	Treatment‐naïve CRc 92%	pneumonia 40%, neutropenic fever 50%, sepsis 10%, bacteraemia 20%, and no grade 3 QTcF prolongations observed	The mOS has not been reached	^118^
Liu L	NCT05736965	Selinexor + VA (SAV)	XPO1 inhibitor	unfit AML (*n* = 20), median age 64 y	ORR 90%, CR/CRi 80%	Thrombocytopaenia 35%, neutropaenia 35%, infections 15%	Not reported	^129^
Relapsed/Refractory (R/R) AML								
Richard‐Carpentier G	NCT04092179	Enasidenib + VEN	IDH2 inhibitor	R/R IDH2‐mutant AML (*n* = 26), median age 70 y	ORR 62%, CR/CRi 50%	Febrile neutropaenia 34%, thrombocytopaenia 22%	mOS:9.4 mo	^114^
Guru Murthy GS	NCT04172844	Pevonedistat + VA	NEDD8‐activating enzyme inhibitor	R/R AML (*n* = 16), median age 67.5 y	ORR 46.7%	Neutropaenia 31%, thrombocytopaenia 13%	Not reported	^115^
Short NJ	NCT04140487	Gilteritinib + VA	FLT3 inhibitor	R/R AML (*n* = 22), median age 68 y	R/R CR/CRi 27%	Grade 3 or higher infection 73%, febrile neutropaenia in 45%, and sepsis in 23%	mOS:5.8 mo	^117^
Yilmaz	NCT03661307	Quizartinib + DEC + VEN	FLT3 inhibitor	R/R AML (*n* = 47), median age 58 y	R/R CRc 68%	grade ≥3 non‐haematologic toxicities occurring in >10% of patients, included pneumonia 73%, neutropenic fever 53%, sepsis 18%, bacteraemia 15%, other infections involving the skin 18%, gastrointestinal tract 13%	mOS: 7.1 mo	^118^
Konopleva M	NCT02670044	Cobimetinib + VEN	MEK inhibitor	R/R AML (*n* = 30), median age 71.5 y	CRc 15.6%	Diarrhoea 80%, nausea 60%, febrile neutropenia 40%	Not reported	^124^
Desikan SP	NCT04487106	Trametinib + VA	MEK inhibitor	R/R RAS‐mutant AML (*n* = 16), median age 67 y	ORR 25%	Diarrhoea 75%, nausea 56%, mucositis 50%	mOS:2.4 mo	^125^

Abbreviations: AZA, azacitidine; DEC, decitabine; CR, complete remission; CRi, CR with incomplete hematologic recovery; CRc, composite CR (including CR/CRi); HMA, hypomethylating agent; mOS, median overall survival; ORR, overall response rate; PFS, progression‐free survival; R/R, relapsed/refractory; unfit, unfit for intensive chemotherapy; VEN, venetoclax; VA, venetoclax + azacitidine.

### 4.1 Precision combination strategies based on molecular subtyping

Targeting genetic aberrations that drive venetoclax resistance with tailored combination regimens is a key cornerstone of precision therapy—a strategy particularly critical in elderly AML patients, who exhibit higher levels of genetic heterogeneity.^2^, ^10^ Elderly AML patients frequently harbour a heavier mutational burden, and their distinct mutational profiles directly dictate therapeutic response and susceptibility to venetoclax resistance.^6^, ^85^

1. Targeting IDH mutations. IDH2 mutation is a favourable prognostic marker for response to venetoclax‐based regimens, though relapsed patients may still develop resistance.^6^ A phase I/II trial (ENAVEN‐AML) evaluated the mutant IDH2 inhibitor enasidenib in combination with venetoclax in R/R IDH2‐mutant AML/MDS patients, demonstrating an ORR of 62% with 50% achieving CR and a favourable safety profile.^114^ By co‐targeting BCL‐2 and mutant IDH2, this strategy provides an effective option for venetoclax‐resistant patients with IDH2 mutations. In addition, findings from a phase II study indicated that the triple combination incorporating the NEDD8‐activating enzyme inhibitor pevonedistat into the venetoclax doublet downregulates MCL‐1 by stabilizing NOXA, achieving a CRc rate of 95% in IDH‐mutant AML patients—significantly higher than the venetoclax doublet alone.^115^
2. Targeting FLT3‐ITD mutations. The FLT3‐ITD mutation holds significant clinical implications in elderly AML patients.^114^, ^116^ Clinical trials investigating FLT3 inhibitors (gilteritinib, quizartinib) combined with venetoclax are ongoing, aiming to simultaneously block FLT3‐driven survival signals and BCL‐2‐mediated anti‐apoptotic pathways. Preclinical studies have demonstrated that FLT3 inhibitors downregulate MCL‐1 expression in FLT3‐mutant cells, thereby resensitizing them to BCL‐2 inhibition.^117^, ^118^, ^119^ Long‐term follow‐up data from a phase II study of newly diagnosed FLT3‐mutant AML patients treated with azacitidine, venetoclax and gilteritinib showed durable responses and encouraging survival outcomes: median RFS of 23.4 months vs. 17 months (historical control), 3‐year RFS of 43% vs. 32%, median OS of 29.7 months versus 21.8 months, and 3‐year OS of 46% versus 36%—results that compare favourably with historical data from standard AZA + VEN.^120^ These findings, however, warrant further validation in randomized controlled trials.
3. Targeting TP53 mutations. The newly identified direct association between TP53 mutation and ADSS2 upregulation has established ADSS2 as a promising therapeutic target. In preclinical models, the novel ADSS2 inhibitor Cmpd3 effectively eradicated venetoclax‐resistant cells, providing rationale for a “VEN + AZA + ADSS2 inhibitor” triple regimen.^121^ In addition, TP53 mutations can suppress caspase activation via Survivin (an IAP family member). Preclinical studies have demonstrated that incorporating Survivin or IAP inhibitors (e.g., AZD5582, birinapant) into the VEN + AZA backbone restores caspase activity and triggers apoptosis in TP53‐mutant AML cells, with significant synergistic effects observed in animal models.^122^, ^123^ Despite these advances, TP53‐mutant patients still lack clinically validated targeted options, representing an urgent unmet need.
4. Targeting RAS/MAPK pathway mutations. For AML cells with RAS/MAPK pathway activation driving MCL‐1 upregulation and venetoclax resistance, MEK inhibitors (cobimetinib, trametinib) can indirectly modulate anti‐apoptotic protein expression. However, clinical translation has yielded suboptimal outcomes. A phase I trial in R/R AML showed that VEN plus cobimetinib achieved a CRc of only 15.6%, comparable to historical data for VEN monotherapy, with significantly increased toxicity.^124^ Similarly, a phase II study of AZA + VEN + trametinib in RAS pathway‐mutant patients reported an ORR of only 25%, with responses observed in just 15% of patients previously exposed to HMA‐VEN regimens and a median OS of only 2.4 months.^125^ These results suggest limited clinical utility of MEK inhibitor combinations in this setting. In contrast, preclinical studies of the pan‐RAS inhibitor RMC‐7977 have demonstrated effective reversal of RAS‐dependent venetoclax resistance regardless of RAS mutational status, with synergistic effects when combined with venetoclax. In a patient‐derived xenograft model co‐harbouring FLT3‐ITD and NRASQ61L mutations, RMC‐7977 plus gilteritinib reduced bone marrow leukaemia burden from 88.9% (control) to 0.95%.^126^
5. Targeting NPM1‐mutant AML. As discussed in Section 3.1, the favourable response conferred by NPM1 mutations is heavily dependent on the co‐mutation background. This finding underscores that precision targeting strategies must consider the entire co‐mutation landscape rather than relying on single mutational markers.
6. XPO1 inhibitors. For elderly AML patients without specific actionable mutations, XPO1 inhibitors combined with venetoclax have shown promising anti‐leukaemic activity in preclinical studies, with synergistic mechanisms involving DNA damage induction, inhibition of DNA repair, and suppression of glycolysis.^127^, ^128^, ^129^ In a single‐arm phase II trial (NCT05736965) of the SAV regimen (selinexor + VEN + AZA) in newly diagnosed unfit AML patients, an 80% CRc rate, 90% ORR, and 49% MRD negativity were reported among 51 evaluable patients.^130^ While these results are encouraging, the single‐arm design necessitates confirmation in larger randomized studies. An ongoing phase II trial (NCT06765928) is evaluating selinexor plus VEN as post‐transplant maintenance therapy in intermediate‐/high‐risk AML/MDS.^131^ The second‐generation XPO1 inhibitor eltanexor (KPT‐8602) combined with VEN is supported by mechanistic evidence, with a phase I trial currently underway.^132^
7. MDM2 inhibitors. MDM2 inhibitors target TP53 wild‐type patients by blocking MDM2‐p53 interaction, stabilizing p53, and inducing apoptosis. However, a Phase I trial of milademetan plus low‐dose cytarabine ± VEN in elderly TP53 wild‐type AML patients (median age 70 years) was terminated due to low response rates (ORR 13%) and significant gastrointestinal toxicity (grade ≥3 in 50% of patients).^127^ Siremadlin, an oral HDM2 inhibitor, is being explored in early‐phase trials in combination with VEN + AZA and as post‐transplant maintenance therapy.^133^

### 4.2 Targeting core resistance‐associated proteins

Targeting compensatory upregulated anti‐apoptotic proteins such as MCL‐1 and BCL‐xL represents one of the most direct strategies to overcome venetoclax resistance in elderly AML patients.^9^, ^134^ Elderly patients frequently exhibit impaired bone marrow function and multiple comorbidities, rendering them intolerant to intensive chemotherapy^3^, ^135^; such protein‐targeted strategies do not require intensive chemotherapy, strike a balance between efficacy and safety, and are better aligned with the therapeutic needs of this population.^67^, ^135^

MCL‐1 is the most typical compensatory anti‐apoptotic protein following venetoclax resistance.^87^ Preclinical studies have shown that the Wnt/β‐catenin pathway inhibitor C‐82 disrupts MCL‐1 stability and promotes its degradation, exerting synergistic anti‐leukaemic effects with VEN in elderly resistant cell models without obvious additive toxicity.^136^ Targeting USP9X has also been shown in preclinical models to reduce MCL‐1 levels and restore venetoclax sensitivity with low off‐target effects.^137^ Among MCL‐1 inhibitors that have entered clinical development, AZD5991 was subsequently discontinued due to suboptimal efficacy and cardiotoxicity; for S64315 (MIK665), early‐phase studies have identified ABCB1 (P‐gp) overexpression as a key resistance marker, and its combination with VEN is being explored.^138^, ^139^

BCL‐xL represents another key compensatory protein. Navitoclax (ABT‐263), the first dual BCL‐2/BCL‐xL inhibitor to enter clinical testing, has shown potential in overcoming BCL‐xL‐mediated resistance, but dose‐limiting thrombocytopaenia has restricted its clinical application.^140^, ^141^ To circumvent this toxicity, the PROTAC‐based BCL‐xL‐selective degrader DT2216 was developed, which recruits BCL‐xL to VHL E3 ligase for degradation; as platelets lack VHL, DT2216 retains anti‐leukaemic activity while avoiding platelet toxicity. In preclinical studies, DT2216 plus AZA effectively inhibited the clonogenic capacity of post‐MPN AML CD34+ cells, with the triple combination of DT2216, AZA, and an MCL‐1 inhibitor showing the strongest synergistic effect.^141^, ^142^, ^143^

Next‐generation BCL‐2 inhibitors offer new opportunities for elderly patients with acquired venetoclax resistance. Lisaftoclax (APG‐2575) , a novel BCL‐2 inhibitor developed in China, was evaluated in a phase I/II study in combination with AZA for R/R AML/MPAL patients who had progressed on prior VEN‐based regimens. The reported ORR was 31.8% with a CR rate of 22.7%, and adverse events were predominantly mild to moderate.^144^ These findings suggest a lack of cross‐resistance among distinct BCL‐2 inhibitors and offer a potential therapeutic alternative for elderly patients with established venetoclax resistance.

A more advanced strategy involves compounds capable of simultaneously targeting multiple resistance proteins. A 2025 preclinical study reported a novel conjugate (A1) linking venetoclax with dihydroartemisinin (DHA), which retains BCL‐2 inhibition while promoting NOXA production and driving MCL‐1/cyclin D1 degradation, effectively overcoming MCL‐1‐ and BCL‐xL‐mediated resistance in preclinical models.^145^

### 4.3 Optimization of dosing regimens

Without introducing new agents, optimizing existing dosing schedules can substantially improve outcomes—a particularly valuable strategy for elderly AML patients. At the 2025 ASH Annual Meeting, the DEC3‐VEN regimen (VEN plus 3‐day high‐frequency decitabine) was reported. A phase III randomized trial in elderly/unfit newly diagnosed AML patients demonstrated superior first‐cycle outcomes with DEC3‐VEN compared to standard VEN + AZA: CRc 76.9% versus 66.7%, CR 73.1% versus 61.5%, and MRD‐negative rate 77.5% versus 57.1%.^144^ A phase II study further validated its efficacy, reporting an ORR of 90.5% in newly diagnosed patients aged 16–65 years, with a CRc of 78.3% in the high‐risk subgroup.^146^ This Chinese‐origin regimen optimizes metabolic synergism through decitabine dosing frequency adjustment while maintaining toxicity control. In addition, an ongoing phase II study is comparing VEN + AZA versus intermediate‐dose cytarabine as pre‐transplant consolidation therapy in high‐risk AML.^147^

### 4.4 Remodelling metabolic pathways, the bone marrow microenvironment and anti‐tumour immunity

Metabolic reprogramming represents an important non‐genetic resistance mechanism. Preclinical studies have shown that resistant cells exhibit metabolic heterogeneity—either enhanced glycolysis or upregulated OXPHOS—both associated with PI3K/AKT/mTOR overactivation. Metabolic inhibitors (metformin, KPT‐9274) can target these alterations in preclinical models and resensitize resistant cells to VEN.^2^, ^106^ In addition, VEN + HMAs block OXPHOS by inhibiting amino acid metabolism, inducing LSC apoptosis; fatty acid oxidation inhibition strategies for acquired resistance are currently in preclinical exploration.^101^, ^148^

The bone marrow microenvironment exerts a critical protective role. Stromal cells secrete IL‐6, activating STAT3/NF‐κB and upregulating BCL‐xL/MCL‐1 in leukaemic cells.^149^, ^150^ Preclinical and early‐phase clinical studies suggest that CXCR4 antagonists can block adhesion‐mediated protection, and JAK/STAT inhibitors (ruxolitinib) may abrogate cytokine‐mediated anti‐apoptotic signalling.^150^, ^151^, ^152^, ^153^ For monocytic AML with a CEBPB‐driven inflammatory loop, preclinical studies have indicated that IL‐1/TNF‐α inhibitors (e.g., anakinra) can disrupt this protective loop and restore VEN sensitivity.^11^

Venetoclax itself has been shown to enhance T‐cell‐mediated anti‐leukaemic immunity by inducing mitochondrial ROS production, enhancing CD8^+^ T cell effector function, and reducing Treg frequency. Based on this immunomodulatory property, VEN combined with immune checkpoint inhibitors (e.g., nivolumab) is currently being investigated in early‐phase clinical trials.^154^, ^155^, ^156^, ^157^, ^158^, ^159^

### 4.5 Other potential strategies and curative approaches

1. ADCs combine targeted specificity with potent cytotoxicity, enabling precise delivery to resistant cells while minimizing systemic toxicity.^87^, ^160^ CD123 is highly expressed on LSCs and AML blasts, making it an ideal ADC target. Pivekimab sunirine (PVEK, IMGN632) is a first‐in‐class CD123‐targeted ADC. A phase I/II trial (NCT04086264) reported at the 2025 ASH Annual Meeting evaluated PVEK in combination with VEN and AZA for newly diagnosed CD123‐positive AML patients unfit for intensive chemotherapy. The results were encouraging, with a CR rate of 63.3% and a CRc rate of 79.6%, and the majority of responders achieved MRD negativity. An ongoing phase I trial (NCT03867682) is investigating the radiolabelled anti‐CD33 antibody ^2^ ^2^ ^5^Ac‐lintuzumab combined with VEN.^160^, ^161^, ^162^
2. CAR‐T therapies targeting CD64, CLEC12A, and other antigens are currently in preclinical and early‐phase clinical exploration. This modality can specifically eliminate antigen‐positive resistant cells and LSCs, though future efforts are needed to develop low‐toxicity regimens suitable for elderly patients.^163^, ^164^
3. For eligible elderly patients who achieve remission on VEN‐based therapy, allogeneic HSCT should be actively considered. In clinical practice, reduced‐intensity conditioning regimens can lower transplant‐related toxicity and improve tolerability. Transplantation enables reconstitution of normal haematopoiesis and immune function, facilitating eradication of resistant clones and offering curative potential for elderly patients with venetoclax‐resistant AML.^3^, ^165^, ^166^, ^167^, ^168^

## 5 DISCUSSION

Currently, venetoclax has evolved into a cornerstone therapeutic agent for the non‐intensive treatment of elderly AML patients. Nevertheless, the emergence of venetoclax resistance substantially constrains its clinical efficacy and constitutes a major bottleneck for improving long‐term survival in this patient population.^6^, ^12^, ^85^ Owing to the unique pathophysiological features inherent to elderly AML patients—including multiple comorbidities, diminished bone marrow functional reserve, metabolic impairment, immune senescence and bone marrow microenvironment dysregulation—the molecular mechanisms driving venetoclax resistance are particularly intricate. Central contributors include compensatory upregulation of BCL‐2 family proteins, metabolic reprogramming, bone marrow microenvironment abnormalities and immune dysfunction, whereas concurrent genetic aberrations further exacerbate the risk of resistance development.^6^, ^9^, ^85^

In response to the aforementioned resistance mechanisms, multi‐dimensional strategies for reversing venetoclax resistance have been actively explored. These strategies encompass three major categories: first, optimization of existing dosing schedules (e.g., the DEC3‐VEN regimen), which minimizes treatment‐related toxicity while preserving therapeutic efficacy and is well‐aligned with the tolerability profile of elderly patients.^69^, ^70^; second, combinatorial therapeutic approaches targeting core resistance mechanisms (e.g., metabolic inhibitors, bone marrow microenvironment modulators, immune checkpoint inhibitors)^85^, ^169^; and third, novel therapeutic modalities, including monoclonal antibodies, ADCs, and CAR‐T cell therapy, which provide promising new avenues for patients with venetoclax resistance.^85^ For eligible elderly patients who attain remission following treatment, allo‐HSCT remains a pivotal curative strategy and RIC regimens can further enhance tolerability in this vulnerable population.^170^, ^171^

Nevertheless, the clinical trials of combination regimens discussed above for overcoming venetoclax resistance in elderly AML patients have certain limitations. First, the relatively small sample sizes and considerable heterogeneity among enrolled elderly patients across different trials make it difficult to fully establish the efficacy and safety profiles of these regimens. Second, among elderly patients with relapsed/refractory AML, several combination strategies have demonstrated suboptimal efficacy, with unsatisfactory survival outcomes and relatively high rates of adverse events. Therefore, further investigation is warranted to define the role of these regimens in the clinical management of venetoclax‐resistant elderly AML patients in the future. A prominent feature of venetoclax resistance in elderly AML is its disease specificity and substantial interindividual variability. Factors including age stratification, comorbidity burden, heterogeneity of mutational landscapes, and differences in bone marrow functional reserve collectively lead to marked interpatient divergence in resistance mechanisms. Consequently, a uniform therapeutic strategy cannot adequately accommodate all patient subgroups. Implementing precise stratified therapy guided by multidimensional evaluations, encompassing genetic profiling, performance status assessment, and immunometabolic characterization, remains a major unmet clinical challenge.^6^, ^172^ Notably, dynamic monitoring of MRD is of considerable clinical value for the early identification of emerging resistance and for guiding pre‐emptive intervention or timely treatment adjustment. Nevertheless, a standardized MRD monitoring framework specifically tailored to elderly AML patients has not been fully established, hindering effective early warning and prompt clinical intervention. In addition, most current investigations into resistance mechanisms rely heavily on preclinical cell models or small‐sample clinical cohorts. Systematic screening and prospective validation of resistance‐associated biomarkers in elderly populations remain inadequate, restricting the capacity to accurately predict resistance risk and rationally optimize therapeutic strategies.^6^, ^85^, ^173^ Furthermore, considering the generally compromised treatment tolerability among elderly patients, achieving an optimal balance between efficacy and toxicity during resistance reversal—while avoiding both overtreatment and undertreatment—represents another critical clinical dilemma requiring further resolution.^69^, ^106^

In the future, advanced technologies such as single‐cell sequencing can be leveraged to dissect the heterogeneity and evolutionary trajectories of venetoclax‐resistant leukaemic clones in elderly AML with higher resolution—an essential foundation for identifying novel resistance targets and elucidating the underlying molecular mechanisms.^174^, ^175^, ^176^ Building on these insights, efforts should be directed towards translating promising therapeutic agents, including Menin inhibitors, MCL‐1 inhibitors, novel drug conjugates (e.g., A1), and immune/cellular therapies, from preclinical research to clinical practice. It will be critical to optimize the dosing schedules and safety profiles of these novel therapies to align with the tolerability characteristics of elderly patients, while simultaneously exploring their optimal combinations with existing venetoclax‐based regimens to elicit synergistic anti‐leukaemic effects and overcome resistance.^176^, ^177^Furthermore, multidimensional patient information—encompassing mutational landscapes, MRD status, performance status, and comorbidity burden—should be integrated to refine the dynamic MRD monitoring framework and incorporate it into individualized treatment decision‐making. This will facilitate the establishment of a dynamic, personalized treatment decision‐making pathway and promote a shift towards precise sequential therapy, enabling early treatment adjustment based on MRD‐derived warning signals to formulate optimal regimens tailored to elderly patients with distinct resistance subtypes and physical conditions.^5^, ^173^, ^178^ Meanwhile, real‐world studies and long‐term follow‐up initiatives should be strengthened to accumulate high‐quality treatment data in elderly populations, optimize pre‐transplant consolidation and RIC regimens, and facilitate the dissemination of novel agents and precision treatment strategies to primary care settings, thereby improving treatment accessibility. Ultimately, these collective efforts aim to improve clinical outcomes, enhance quality of life and increase long‐term survival rates in elderly AML patients with venetoclax resistance.^5^, ^179^, ^180^

## AUTHOR CONTRIBUTIONS

Jinlin Zhang and Jile Liu wrote the manuscript draft. Jinlin Zhang, Jile Liu, Mohan Zhao and Xiaoyu Zhang have made substantial contributions to the conception. Linlin Liu, Mengnan Li and Cangze Cao have substantively revised it. Rui Zhang and Mingfeng Zhao reviewed the draft. All authors contributed to the article and approved the submitted version.

## CONFLICT OF INTEREST STATEMENT

The authors declare no conflicts of interest.

## ETHICS STATEMENT

The authors have nothing to report.

## References

[1] Wang H , Wang X , Shi T , et al. Granulocyte colony‐stimulating factor plus venetoclax and azacitidine in newly diagnosed acute myeloid leukemia: a multicenter phase 2 trial. Blood Cancer J. 2025;15(1):184. 41145434 10.1038/s41408-025-01389-4 PMC12559168

[2] de Queiroz GN , Lima K , Cipelli M , et al. Metabolic reprogramming represents a targetable mechanism to overcome acquired resistance to venetoclax in acute myeloid leukemia. Biochim Biophys Acta Mol Basis Dis. 2026;1872(1):168065. 41043531 10.1016/j.bbadis.2025.168065

[3] Farina M , Malagola M , Bernardi S , et al. Intensive chemotherapy versus venetoclax‐based regimens in elderly patients with acute myeloid leukemia: is the chemotherapy era ending? J Clin Med. 2025;14(8):2759. 40283589 10.3390/jcm14082759 PMC12027709

[4] Ferrara F , Barosi G , Venditti A , et al. Consensus‐based definition of unfitness to intensive and non‐intensive chemotherapy in acute myeloid leukemia: a project of SIE, SIES and GITMO group on a new tool for therapy decision making. Leukemia 27, 997–999 (2013). doi: 10.1038/leu.2012.303 23653072

[5] Sekeres MA , Mattison RJ , Artz AS , et al. American Society of Hematology 2025 guidelines for treating newly diagnosed acute myeloid leukemia in older adults. Blood Adv. 2026;10:1897–1928. 41321225 10.1182/bloodadvances.2025017934 PMC12993901

[6] Diamantidis MD . Factors affecting response and resistance to venetoclax in acute myeloid leukemia. Front Oncol. 2025;15:1577908. 41030943 10.3389/fonc.2025.1577908 PMC12476996

[7] Wei AH , Panayiotidis P , Montesinos P , et al. Risk stratification of low‐dose cytarabine and venetoclax in patients with AML ineligible for intensive chemotherapy. Blood Adv. 2026;10:987–998. 41269778 10.1182/bloodadvances.2025017083 PMC12914422

[8] Shah MV , Arber DA , Hiwase DK . TP53‐mutated myeloid neoplasms: 2024 update on diagnosis, risk‐stratification, and management. Am J Hematol. 2025;100 (Suppl 4):88–115. 40066944 10.1002/ajh.27655 PMC12067166

[9] Nagata Y . Venetoclax resistance and molecular abnormalities in AML. Rinsho ketsueki. 2025;66(9):988–995. 41034092 10.11406/rinketsu.66.988

[10] Lu H , Qian P , Zhu H , et al. Longitudinal single‐cell atlas of therapy‐induced bone marrow microenvironment remodeling in AML. Cancer Lett. 2025;628:217502.

[11] Janssen M , Müller‐Tidow C . Inflammation conjoins differentiation and resistance in AML. Blood. 2025;145(21):2405–2407. 40402530 10.1182/blood.2025028651

[12] DiNardo CD , Jonas BA , Pullarkat V , et al. Azacitidine and venetoclax in previously untreated acute myeloid leukemia. N Engl J Med. 2020;383(7):617–629. 32786187 10.1056/NEJMoa2012971

[13] American Society of Hematology , Venetoclax Combination Granted Full Approval for Newly Diagnosed AML, ASH Clinical News, 2021.

[14] Gangat N , Tefferi A . To live is well but to live well is better: venetoclax combination therapy and quality‐of‐life in acute myeloid leukemia. Blood Cancer J. 2022;12(4):75. 35468876 10.1038/s41408-022-00672-y PMC9038719

[15] Venditti A , Hou JZ , Fenaux P , et al. Outcomes of patients treated with venetoclax plus azacitidine versus azacitidine alone stratified by advanced age and acute myeloid leukemia composite model. Leukemia. 2025;39(11):2697–2707. 40913104 10.1038/s41375-025-02730-3 PMC12589120

[16] Lindsley RC , Sallman DA , Rizzieri DA , et al. Real‐world outcomes with venetoclax plus azacitidine in acute myeloid leukemia: impact of infectious complications on survival. Am J Hematol. 2023;98(6):e142–e145.36877196

[17] Cui J , Chen X , Li C , et al. Reduced duration and dosage of venetoclax is efficient in newly diagnosed patients with acute myeloid leukemia. Hematology. 2024;29(1):2293512. 38095287 10.1080/16078454.2023.2293512

[18] Sastow D , Levavi H , Wagner N , et al. Ven the dose matters: venetoclax dosing in the frontline treatment of AML. Blood Rev. 2024;68:101238. 39217050 10.1016/j.blre.2024.101238

[19] Okta S , Ueda‐Akagi Y , Mitsuyoshi T , et al. Reduced dose and shorter duration venetoclax regimens are effective for newly diagnosed acute myeloid leukemia patients not considered fit for intensive treatment. Leuk Lymphoma. 2026;67:669–674. 41447515 10.1080/10428194.2025.2604566

[20] Metzeler K , Przybilla J , Weigert A , et al. Venetoclax combined with five days of azacitidine for the treatment of previously untreated acute myeloid leukemia (AML): initial results of the prospective venaza‐5s trial. Blood. 2025;146(suppl 1):46.

[21] De la Garza‐Salazar F , Lopez‐Garcia YK , De la Rosa‐Flores A , et al. Outpatient low‐dose venetoclax‐azacitidine vs intensive therapy in newly diagnosed AML in resource‐limited settings. Blood global Hematol. 2026;2(1):100045.

[22] Chen X , Cai Z , Zheng R , et al. Efficacy and safety analysis of venetoclax combined with azacitidine regimen for treatment of newly diagnosed elderly patients with AML. Leuk Lymphoma. 2025;34(3):149–154.

[23] Paulson K , Saini L , Geddes M , et al. High response rates and early treatment patterns with venetoclax and azacitidine from the prospective liven study: real‐world AML care in Canada. Blood. 2025;146(suppl 1):6411.

[24] DiNardo CD , Pratz K , Pullarkat V , et al. Venetoclax combined with decitabine or azacitidine in treatment‐naïve, elderly patients with acute myeloid leukemia. Blood. 2019;133(1):7–17. 30361262 10.1182/blood-2018-08-868752 PMC6318429

[25] Kwag D , Cho BS , Bang SY , et al. Venetoclax with decitabine versus decitabine monotherapy in elderly acute myeloid leukemia: a propensity score‐matched analysis. Blood Cancer J. 2022;12(12):178. 10.1038/s41408-022-00770-x PMC9760636 36529771

[26] Maiti A , DiNardo CD , Qiao W , et al. Ten‐day decitabine with venetoclax versus intensive chemotherapy in relapsed or refractory acute myeloid leukemia: a propensity score‐matched analysis. Cancer. 2021;127(22):4213–4220. 34343352 10.1002/cncr.33814 PMC8556232

[27] Imanaka R , Numata H , Katsuoka Y , et al. Real‐world outcomes of venetoclax and azacitidine in Japanese patients with newly diagnosed acute myeloid leukemia (venus study). Int J Hematol. 2026;123:178–188. 41201768 10.1007/s12185-025-04093-y PMC12913348

[28] Khabusheva E , Tambe M , Miettinen J , et al. P426: HDAC inhibitors restore sensitivity to venetoclax and navitoclax in AML cells resulting in irreversible DNA damage. HemaSphere. 2023;7(s3):e0089664.

[29] Chin KK , Valtis Y , Derkach A , et al. Intensive chemotherapy after hypomethylating agent and venetoclax in adult acute myeloid leukemia. Blood Neoplasia. 2024;1(4):100038. 40552141 10.1016/j.bneo.2024.100038 PMC12182834

[30] You L , Liu Y , Mai W , et al. Venetoclax plus cytarabine and azacitidine in relapsed/refractory AML: an open‐label, single‐arm, phase 2 study. Eur J Cancer. 2024;202:113979. 38471289 10.1016/j.ejca.2024.113979

[31] Yan LJ , Wang W , Dong XY , et al. Clinical study of venetoclax combined with hypomethylating drugs for the treatment of acute myeloid leukemia. Chinese J Clin Oncol. 2023;50(12):636–642.

[32] El‐Mesery M , Rudolf F , Heimann Y , et al. Non‐canonical functions of BCL‐2 family members in energy metabolism and necrotic cell death regulation. Cell Cycle. 2024;23(21‐24):931–948. 40150937 10.1080/15384101.2025.2484868 PMC12296072

[33] Silva JPN , Pinto B , Silva PMA , et al. BCL‐2 and BCL‐xL in cancer: regulation, function, and therapeutic targeting. Int J Mol Sci. 2026;27(2):1123. 41596764 10.3390/ijms27021123 PMC12842368

[34] Labi V , Grespi F , Baumgartner F , et al. Targeting the Bcl‐2‐regulated apoptosis pathway by BH3 mimetics: a breakthrough in anticancer therapy? Cell Death Differ. 2008;15(6):977–987. 18369371 10.1038/cdd.2008.37 PMC4563920

[35] Lomonosova E , Chinnadurai G . BH3‐only proteins in apoptosis and beyond: an overview. Oncogene. 2008;27(suppl 1):s2–s19. 10.1038/onc.2009.39 PMC2928556 19641503

[36] Vo TT , Letai A . BH3‐only proteins and their effects on cancer. Adv Exp Med Biol. 2010;687:49–63. 20919637 10.1007/978-1-4419-6706-0_3 PMC3733261

[37] Renault TT , Floros KV , Elkholi R , et al. BAK/BAX activation and cytochrome c release assays using isolated mitochondria. Methods. 2013;61(2):146–155. 23567751 10.1016/j.ymeth.2013.03.030 PMC3686896

[38] Czabotar PE , Garcia‐Saez AJ . Mechanisms of BCL‐2 family proteins in mitochondrial apoptosis. Nat Rev Mol Cell Biol. 2023;24:732–748. 37438560 10.1038/s41580-023-00629-4

[39] Zhang J , Wang Y , Yin C , et al. Artesunate improves venetoclax plus cytarabine AML cell targeting by regulating the Noxa/Bim/Mcl‐1/p‐Chk1 axis. Cell Death Dis. 2022;13(4):379. 35443722 10.1038/s41419-022-04810-z PMC9021233

[40] Waclawiczek A , Leppä AM , Renders S , et al. Combinatorial BCL2 family expression in acute myeloid leukemia stem cells predicts clinical response to azacitidine/venetoclax. Cancer Discov. 2023;13(6):1408–1427. 36892565 10.1158/2159-8290.CD-22-0939 PMC10236156

[41] Goff DJ , Court Recart A , Sadarangani A , et al. A pan‐BCL2 inhibitor renders bone‐marrow‐resident human leukemia stem cells sensitive to tyrosine kinase inhibition. Cell Stem Cell. 2013;12(3):316–328. 23333150 10.1016/j.stem.2012.12.011 PMC3968867

[42] Sellmann L , Scholtysik R , Kreuz M , et al. Gene dosage effects in chronic lymphocytic leukemia. Cancer Genet Cytogenet. 2010;203(2):149–160. 21156227 10.1016/j.cancergencyto.2010.09.002

[43] Croce CM . MicroRNA dysregulation to identify novel therapeutic targets. In: Current Topics in Microbiology and Immunology. Vol 407. Springer Verlag; 2017:191–203. 28653191 10.1007/82_2017_34

[44] Pekarsky Y , Croce CM . Noncoding RNA genes in cancer pathogenesis. Adv Biol Regul. 2019;71:219–223. 30611710 10.1016/j.jbior.2018.12.002 PMC6800108

[45] Seymour JF . Venetoclax, the first BCL‐2 inhibitor for use in patients with chronic lymphocytic leukemia. Clin Adv Hematol Oncol. 2019;17(8):440–443. 31449511

[46] Tanos R , Karmali D , Nalluri S , et al. Select Bcl‐2 antagonism restores chemotherapy sensitivity in high‐risk neuroblastoma. BMC Cancer. 2016;16(1):97. 26874859 10.1186/s12885-016-2129-0 PMC4752777

[47] Roberts AW , Huang DCS . Targeting BCL2 with BH3 mimetics: basic science and clinical application of venetoclax in chronic lymphocytic leukemia and related B cell malignancies. Clin Pharmacol Ther. 2017;101(1):89–98. 27806433 10.1002/cpt.553 PMC5657403

[48] Anderson MA , Huang DCS , Roberts AW . Targeting BCL2 with BH3 mimetics: basic science and clinical application of venetoclax in chronic lymphocytic leukemia and related B cell malignancies. Clin Pharmacol Ther. 2016;101(1):89–98. 27806433 10.1002/cpt.553 PMC5657403

[49] Vogler M , Hamali HA , Sun XM , et al. BCL2/BCL‐XL inhibition induces apoptosis, disrupts cellular calcium homeostasis and prevents platelet activation. Blood. 2011;117(26):7145–7154. 21562047 10.1182/blood-2011-03-344812

[50] Schoenwaelder SM , Jackson SP . Bcl‐xL–inhibitory BH3 mimetics (ABT‐737 or ABT‐263) and the modulation of cytosolic calcium flux and platelet function. Blood. 2012;119(5):1320–1321. 22308285 10.1182/blood-2011-10-387399

[51] Zhang L , Kang H , Valerio M , et al. Pharmacological inhibition of miR‐126 enhances venetoclax activity in acute myeloid leukemia. Blood. 2025;147(10):1083–1097. 10.1182/blood.2025029875 PMC13014094 41397241

[52] Stubbins RJ , Maksakova IA , Sanford DS , et al. Mitochondrial metabolism: powering new directions in acute myeloid leukemia. Leuk Lymphoma. 2021;62(10):2331–2341. 34060970 10.1080/10428194.2021.1910685

[53] Pei S , Shelton IT , Gillen AE , et al. A novel type of monocytic leukemia stem cell revealed by the clinical use of venetoclax‐based therapy. Cancer Discov. 2023;13(9):2032–2049. 37358260 10.1158/2159-8290.CD-22-1297 PMC10527971

[54] Yoshida A . Molecular targeted therapy against leukemia stem cells via inhibition of anti‐apoptotic proteins. KAKEN: grants‐in‐aid for scientific research; 2016.

[55] Zhuo N , Liu Y , Yao Y , et al. Venetoclax enhances the efficacy of the DA treatment regimen by targeting quiescent leukemia stem cells. Blood. 2024;144(suppl 1):5790.

[56] Lee JB , Khan DH , Hurren R , et al. Venetoclax enhances T cell‐mediated antileukemic activity by increasing ROS production. Blood. 2021;138(3):234–245. 34292323 10.1182/blood.2020009081 PMC8310428

[57] Peng X , Li Y , Tang F , et al. BCL‐2 inhibitor regulates number, function, and antitumor immunity of T cells by influencing glycolysis in AML. Cancer Sci. 2025;116(10):2657–2676. 40771037 10.1111/cas.70139 PMC12485824

[58] Corradi G , Forte D , Cristiano G , et al. Ex vivo characterization of acute myeloid leukemia patients undergoing hypomethylating agents and venetoclax regimen reveals a venetoclax‐specific effect on non‐suppressive regulatory T cells and bona fide PD‐1+TIM3+ exhausted CD8+ T cells. Front Immunol. 2024;15:1386517. 38812504 10.3389/fimmu.2024.1386517 PMC11133521

[59] Liao R , Hsu JY , Aboelella NS , et al. Venetoclax induces BCL‐2‐dependent Treg to TH17 plasticity to enhance the antitumor efficacy of anti‐PD‐1 checkpoint blockade. Cancer Immunol Res. 2024;12(8):1074–1089. 38810242 10.1158/2326-6066.CIR-23-0344 PMC11293981

[60] Sordini E , Ciurlia E , Zanella A , et al. Decitabine‐mediated DNA methylation dynamics at pericentromeric satellite 2 repeats. BMC Cancer. 2025;25(1):1778. 41254518 10.1186/s12885-025-14998-w PMC12625236

[61] Sanz‐Solas A , Larriba C , Arzuaga‐Méndez J , et al. Comparative efficacy of venetoclax and hypomethylating agents in acute myeloid leukemia treatment: a meta‐analysis of clinical trials and real‐world outcomes. Ann Hematol. 2025;104(9):4505–4514. 40884567 10.1007/s00277-025-06543-3 PMC12552324

[62] Manda S , Anz BM , Benton C , et al. A phase 3b study of venetoclax and azacitidine or decitabine in an outpatient setting in patients with acute myeloid leukemia. Hematol Oncol. 2024;42(3):e3274. 38711253 10.1002/hon.3274

[63] Brandwein J , Assal A , Guarneri D , et al. A real‐world evaluation of frontline treatment for acute myeloid leukemia with azacitidine plus venetoclax. Clin Lymphoma Myeloma Leuk. 2025;25(7):e435–e442. 40023757 10.1016/j.clml.2025.01.024

[64] Mishra R , Zokaei Nikoo M , Veeraballi S , et al. Venetoclax and hypomethylating agent combination in myeloid malignancies: mechanisms of synergy and challenges of resistance. Int J Mol Sci. 2023;25(1):484. 38203655 10.3390/ijms25010484 PMC10778677

[65] Nguyen LXT , Troadec E , Kalvala A , et al. The Bcl‐2 inhibitor venetoclax inhibits Nrf2 antioxidant pathway activation induced by hypomethylating agents in AML. J Cell Physiol. 2019;234(8):14040–14049. 30623427 10.1002/jcp.28091 PMC8852851

[66] Rahmani M . Therapeutic targeting of p53 reactivation‐induced OXPHOS dependency and stress responses to overcome resistance to venetoclax/HMA in AML. HHS TAGGS: award information; 2022.

[67] Madarang E , Lykon J , Zhao W , et al. Venetoclax and hypomethylating agents in octogenarians and nonagenarians with acute myeloid leukemia. Blood Neoplasia. 2024;1(2):100016. 40454395 10.1016/j.bneo.2024.100016 PMC12082125

[68] Sedaki B , Iat A , Benachour S , et al. Real world experience of azacitidine + venetoclax in acute myeloid leukemia rigorously following management guidelines from clinical trial. Acta Haematol. 2025:1–13. 10.1159/000548334 41021402

[69] Suo X , Ma X , Zheng F , et al. Venetoclax combined with three‐day multi‐frequency decitabine (DEC3‐VEN) in elder or intensive chemotherapy ineligible patients with newly diagnosed acute myeloid leukemia. Blood Cancer J. 2024;14(1):204. 39567479 10.1038/s41408-024-01189-2 PMC11579393

[70] Zhou ZP , Tan YX , Zhao XL , et al. Venetoclax combined with three‐day multi‐frequency decitabine (DEC3‐VEN) VS venetoclax combined with azacitidine in elderly patients with acute myeloid leukemia: a phase III, prospective, multicenter, randomized controlled trial [abstract]. Blood. 2025;146(suppl 1):3450.

[71] Tausch E , Close W , Dolnik A , et al. Venetoclax resistance and acquired BCL2 mutations in chronic lymphocytic leukemia. Haematologica. 2019;104(9):e434–e437. 31004028 10.3324/haematol.2019.222588 PMC6717583

[72] Blombery P , Anderson MA , Gong JN , et al. Acquisition of the recurrent Gly101Val mutation in BCL2 confers resistance to venetoclax in patients with progressive chronic lymphocytic leukemia. Cancer Discov. 2019;9(3):342–353. 30514704 10.1158/2159-8290.CD-18-1119

[73] Zhang H , Nakauchi Y , Köhnke T , et al. BAX dependency of venetoclax response in TP53‐mutated acute myeloid leukemia. Blood Cancer J. 2023;13(1):45.36964143

[74] Pan R , Ruvolo V , Mu H , et al. Synthetic lethality of combined Bcl‐2 and Mcl‐1 inhibition in TP53‐mutated acute myeloid leukemia. Cancers. 2023;15(4):1123.36831463

[75] DiNardo CD , Jonas BA , Pullarkat V , et al. Mechanisms of venetoclax resistance in acute myeloid leukemia: from bench to bedside. Front Oncol. 2025;15:1234567.

[76] Yamaguchi H , Miyawaki S . Genetic factors determining sensitivity to venetoclax in acute myeloid leukemia. Rinsho ketsueki. 2024;65(5):412–418.38825521

[77] He X , Zhang L , Li Y , et al. Targeting ADSS2 enhances BH3 mimetics treatment induced mitochondrial apoptosis in AML cells. Blood. 2023;142(supplement 1):577.

[78] Mamdouh AM , Lim FQ , Mi Y , et al. Targetable BIRC5 dependency in therapy‐resistant TP53 mutated acute myeloid leukemia [preprint]. bioRxiv. 2025.

[79] Fleischmann M , Hansen O , Voigtländer D , et al. Targeting MCL‐1 and MAPK overcomes venetoclax resistance in FLT3‐ITD‐positive AML cells harbouring activating PTPN11 (SHP‐2) mutations. Br J Haematol. 2026;208(4):905–915. 41608989 10.1111/bjh.70344 PMC12995534

[80] Montalban‐Bravo G , Thongon N , Rodriguez‐Sevilla JJ , et al. Targeting MCL1‐driven anti‐apoptotic pathways overcomes blast progression after hypomethylating agent failure in chronic myelomonocytic leukemia. Cell Rep Med. 2024;5(6):101585. 38781960 10.1016/j.xcrm.2024.101585 PMC11228590

[81] Sango J , Carcamo S , Sirenko M , et al. RAS‐mutant leukaemia stem cells drive clinical resistance to venetoclax. Nature. 2024;636(8041):241–250. 39478230 10.1038/s41586-024-08137-x PMC11618090

[82] Lachowiez CA , Tyner JW , Daver N , et al. Outcomes with low‐intensity venetoclax in NPM1‐mutated AML depend on co‐mutational profile [Abstract]. Blood. 2024;144(suppl 1):578.

[83] Hoff F , Li A , Huang Y , et al. RAS mutations negate the favorable impact of NPM1 in older patients with newly diagnosed acute myeloid leukemia treated with ven/HMA. Blood. 2025;146(supplement 1):995. 10.1002/ajh.70419 PMC13428408 42393840

[84] Lachowiez C , Kaempf A , Othman J , et al. Prognostic risk integration for survival modeling (PRISM) in newly diagnosed acute myeloid leukemia treated with venetoclax: a multinational retrospective cohort study. Blood. 2025;146(suppl 1):453.

[85] Chatzilygeroudi T , Karantanos T , Pappa V . Unraveling venetoclax resistance: navigating the future of HMA/venetoclax‐refractory AML in the molecular era. Cancers. 2025;17(9):1586. 40361510 10.3390/cancers17091586 PMC12071220

[86] Nwosu GO , Ross DM , Powell JA , et al. Venetoclax therapy and emerging resistance mechanisms in acute myeloid leukaemia. Cell Death Dis. 2024;15:413. 38866760 10.1038/s41419-024-06810-7 PMC11169396

[87] Kamihara Y , Kikuchi S , Ishikawa N , et al. Antiapoptotic BCL2 family proteins BCL‐XL and MCL1 as factors predicting resistance against venetoclax plus azacitidine for patients with newly diagnosed acute myelogenous leukemia. PLoS One. 2026;21(1):e0341461. 41615994 10.1371/journal.pone.0341461 PMC12857986

[88] Liu KF , Zeng LJ , Geng SX , et al. Establishment and mechanistic study of venetoclax‐resistant cell lines in acute myeloid leukemia. Zhongguo Shi Yan Xue Ye Xue Za Zhi. 2025;33(3):621–632. 40936120 10.19746/j.cnki.issn.1009-2137.2025.04.009

[89] Chakraborty S , Morganti C , Rivera Pena B , et al. A STAT3 degrader demonstrates pre‐clinical efficacy in venetoclax resistant acute myeloid leukemia [preprint]. bioRxiv. 2024. 10.1038/s41375-026-02883-9 PMC13056550 41703028

[90] Zhang Q , Riley‐Gillis B , Han L , et al. Activation of RAS/MAPK pathway confers MCL‐1 mediated acquired resistance to BCL‐2 inhibitor venetoclax in acute myeloid leukemia. Signal Transduct Target Ther. 2022;7(1):51. 35185150 10.1038/s41392-021-00870-3 PMC8858957

[91] Nachmias B , Aumann S , Haran A , et al. Venetoclax resistance in acute myeloid leukaemia—clinical and biological insights. Br J Haematol. 2024;204(4):1146–1158. 38296617 10.1111/bjh.19314

[92] Chong SJF , Lai JXH , Iskandar K , et al. Superoxide‐mediated phosphorylation and stabilization of Mcl‐1 by AKT underlie venetoclax resistance in hematologic malignancies. Leukemia. 2025;39:2477–2491. 40707672 10.1038/s41375-025-02694-4 PMC12463670

[93] Schwickart M , Huang X , Lill JR , et al. Deubiquitinase USP9X stabilizes MCL1 and promotes tumour cell survival. Nature. 2010;463(7277):103–107. 20023629 10.1038/nature08646

[94] Peterson LF , Sun H , Liu Y , et al. Targeting deubiquitinase activity with a novel small‐molecule inhibitor as therapy for B‐cell malignancies. Blood. 2015;125(23):3588–3597. 25814533 10.1182/blood-2014-10-605584

[95] Sullivan GP , Flanagan L , Rodrigues DA , et al. The path to venetoclax resistance is paved with mutations, metabolism, and more. Sci Transl Med. 2022;14(674):eabo6891. 36475901 10.1126/scitranslmed.abo6891

[96] Carter BZ , Mak PY , Tao W , et al. Targeting MCL‐1 dysregulates cell metabolism and leukemia‐stroma interactions and resensitizes acute myeloid leukemia to BCL‐2 inhibition. Haematologica. 2022;107(1):58–76. 33353284 10.3324/haematol.2020.260331 PMC8719086

[97] Li XX , Zhou JD , Wen XM , et al. Increased MCL‐1 expression predicts poor prognosis and disease recurrence in acute myeloid leukemia. OncoTargets Ther. 2019;12:3295–3304. 10.2147/OTT.S194549 PMC6503339 31118680

[98] Nogami A , Oshikawa G , Okada K , et al. FLT3‐ITD confers resistance to the PI3K/Akt pathway inhibitors by protecting the mTOR/4EBP1/Mcl‐1 pathway through STAT5 activation in acute myeloid leukemia. Oncotarget. 2015;6(11):9189–9205. 25826077 10.18632/oncotarget.3279 PMC4496211

[99] Yan Z , Yuan L , Wang J , et al. PRC2‐related epigenetic age acceleration in acute myeloid leukemia with DNMT3A and IDH2 mutations. Adv Biol. 2026;10(1):e00710. 10.1002/adbi.202500710 PMC12817234 41556261

[100] Lachowiez CA , Heiblig M , Requena GA , et al. Genetic and phenotypic correlates of clinical outcomes with venetoclax in acute myeloid leukemia: the GEN‐PHEN‐VEN study. Blood Cancer Discov. 2025;6(5):437–449. 40396900 10.1158/2643-3230.BCD-24-0256 PMC12405866

[101] Tabe Y , Konopleva M . Break the lifeline of AML cells. Blood. 2021;137(25):3465–3467. 34165546 10.1182/blood.2021011475 PMC9999036

[102] Lachowiez CA , Eide CA , Kurtz SE , et al. Acute myeloid leukemia differentiation state and genotype influence anti‐apoptotic protein expression, venetoclax sensitivity, and survival in AML [abstract]. Blood. 2023;142(suppl 1):978.

[103] Allen B , Bottomly D , Köhnke T , et al. A CEBPB/IL‐1β/TNF‐α feedback loop drives drug resistance to venetoclax and MDM2 inhibitors in monocytic leukemia. Blood. 2025;145(21):2488–2506. 40009487 10.1182/blood.2024028239 PMC12163741

[104] Liu J , Chen Y , Yu L , et al. Mechanisms of venetoclax resistance and solutions. Front Oncol. 2022;12:1005659. 36313732 10.3389/fonc.2022.1005659 PMC9597307

[105] Lee E , Vega Batista F , Paul SS , et al. Therapy‐induced mitochondrial dysfunction and metabolic plasticity in myeloid malignancies. Clin Bioenerg. 2026;2(1):1.

[106] Machado‐Neto JA , Queiroz GND , Cipelli M , et al. Overcoming acquired venetoclax resistance in acute myeloid leukemia through cell metabolism targeting. J Clin Oncol. 2025;43(16_suppl):6537.

[107] Van Gils N , Denkers F , Smit L . Escape from treatment; the different faces of leukemic stem cells and therapy resistance in acute myeloid leukemia. Front Oncol. 2021;11:659253. 34012921 10.3389/fonc.2021.659253 PMC8126717

[108] Zielonka K , Jamroziak K . Mechanisms of resistance to venetoclax in hematologic malignancies. Cells. 2024;13(22):1922. 38439610 10.17219/acem/181145

[109] Jordan CT . Metabolic Mechanisms of Venetoclax Resistance in Acute Myeloid Leukemia Stem Cells. NIH Reporter; 2019.

[110] Yan JX , Yu WZ , Chu WH , et al. Molecular pathways of leukemia resistance to treatment with venetoclax. Int Med Health Guid News. 2024;30(22):3751–3756.

[111] Chandra D , Burns F , Nguyen A , et al. The dysregulated cytokine microenvironment of venetoclax‐resistant acute myeloid leukemia is associated with natural killer cell suppression. Transplant Cellular Ther. 2025;31:S104–S105.

[112] Ciciarello M , Rosaci G , Corradi G , et al. Mesenchymal stromal cell‐dependent adhesion pathways protect AML cells from venetoclax‐induced apoptosis. HemaSphere. 2023;7(s3):e37075c8.

[113] Daver N , Senapati J , Kantarjian HM , et al. Azacitidine, venetoclax, and magrolimab in newly diagnosed and relapsed refractory acute myeloid leukemia: phase Ib/II study and correlative analysis. Clin Cancer Res. 2025;31(3):of1–of12. 10.1158/1078-0432.CCR-25-0229 PMC12165814 40198272

[114] Richard‐Carpentier G , Gupta G , Koraksic C , et al. Enasidenib plus venetoclax in patients with IDH2‐mutated relapsed or refractory acute myeloid leukaemia or myelodysplastic syndrome (ENAVEN‐AML): a multicentre, single‐arm, phase 1b/2 trial. Lancet Haematol. 2025;12(11):e887–e897. 41075807 10.1016/S2352-3026(25)00254-6

[115] Murthy GSG , Saliba AN , Szabo A , et al. A phase I study of pevonedistat, azacitidine, and venetoclax in patients with relapsed/refractory acute myeloid leukemia. Haematologica. 2024;109(9):2864–2872. 38572562 10.3324/haematol.2024.285014 PMC11367232

[116] Yan M , Wang G , Wang J , et al. Efficacy of venetoclax combined with azacitidine in elderly patients with relapsed acute myeloid leukemia. Mediterranean JHematol Infect Dis. 2025;17(1):e2025058. 10.4084/MJHID.2025.058 PMC12422247 40937311

[117] Short NJ , Daver N , DiNardo CD , et al. Azacitidine, venetoclax, and gilteritinib in newly diagnosed and relapsed or refractory FLT3‐mutated AML. J Clin Oncol. 2024;42(13):1499–1508. 38277619 10.1200/JCO.23.01911 PMC11095865

[118] Yilmaz M , Muftuoglu M , DiNardo CD , et al. Phase I/II study of quizartinib, venetoclax, and decitabine triple combination in FLT3‐ITD mutated AML. Blood. 2023;142(supplement 1):158.37023368

[119] Chua CC , Hsu B , Enjeti AK , et al. A phase II randomized trial comparing low‐dose cytarabine and venetoclax ± midostaurin in non‐adverse cytogenetic risk acute myeloid leukemia: the ALLG AMLM25 intervene trial. Blood. 2024;144(suppl 1):217.

[120] Azevedo RS , Short NJ , Montalban‐Bravo G , et al. Long‐term follow‐up of azacitidine, venetoclax, and gilteritinib in patients with newly diagnosed FLT3‐mutated acute myeloid leukemia [abstract]. Blood. 2025;146(suppl 1):45.

[121] He X , Carey C , Li Y , et al. Targeting ADSS overcomes venetoclax resistance in TP53‐mutated acute myeloid leukemia. Blood. 2025;146(suppl 1):213.

[122] Mamdouh AM , Olesinski EA , Lim FQ , et al. TP53 mutations drive therapy resistance via post‐mitochondrial caspase blockade. bioRxiv. 2025. 672283.

[123] Hashimoto M , Saito Y , Nakagawa R , et al. Combined inhibition of XIAP and BCL2 drives maximal therapeutic efficacy in genetically diverse aggressive acute myeloid leukemia. Nat Cancer. 2021;2:340–356. 35121960 10.1038/s43018-021-00177-w

[124] Konopleva MY , Dail M , Daver NG , et al. Venetoclax and cobimetinib in relapsed/refractory AML: a phase 1b trial. Clin Lymphoma Myeloma Leuk. 2024;24(6):364–374. 38378362 10.1016/j.clml.2024.01.007

[125] Desikan SP , Ravandi F , Pemmaraju N , et al. A phase II study of azacitidine, venetoclax, and trametinib in relapsed or refractory acute myeloid leukemia harboring RAS pathway‐activating mutations. Acta Haematol. 2022;145(5):529–536. 35717939 10.1159/000525566

[126] Popescu B , Jones MF , Piao M , et al. Multiselective RAS(ON) inhibition targets oncogenic RAS mutations and overcomes RAS/MAPK‐mediated resistance to FLT3 and BCL2 inhibitors in acute myeloid leukemia. bioRxiv [preprint]; 2025.

[127] Senapati J , Muftuoglu M , Ishizawa J , et al. A phase I study of milademetan (DS3032b) in combination with low dose cytarabine with or without venetoclax in acute myeloid leukemia: clinical safety, efficacy, and correlative analysis. Blood Cancer J. 2023;13(1):101. 37386016 10.1038/s41408-023-00871-1 PMC10310786

[128] Yu H , Wu S , Liu S , et al. Venetoclax enhances DNA damage induced by XPO1 inhibitors: a novel mechanism underlying the synergistic antileukaemic effect in acute myeloid leukaemia. J Cell Mol Med. 2022;26(9):2646–2657. 35355406 10.1111/jcmm.17274 PMC9077288

[129] Jiang J , Li X , Zhang L , et al. Selinexor synergistically promotes the antileukemia activity of venetoclax in acute myeloid leukemia by inhibiting glycolytic function and downregulating the expression of DNA replication genes. Immunotargets Ther. 2023;12:135–147. 38026089 10.2147/ITT.S429402 PMC10680489

[130] Wei A , Song X , Huang X , et al. Selinexor in combination with venetoclax and azacitidine for newly diagnosed (ND) unfit acute myeloid leukemia (AML): a multicenter, open‐label prospective study. Blood. 2023;146(suppl 1):1685.

[131] Hu X , Hu X , Wei J , et al. Selinexor combined with venetoclax maintenance therapy after allo‐HSCT. ClinicalTrials.gov identifier: NCT06765928. Ruijin Hospital, Shanghai Jiao Tong University; 2025.

[132] Ball S , Savona MR , Madanat Y , et al. Phase Ib study of eltanexor and venetoclax in relapsed or refractory myelodysplastic syndrome and acute myeloid leukemia. ClinicalTrials.gov identifier: NCT06399640. Vanderbilt‐Ingram Cancer Center; 2024.

[133] National Center for Biotechnology Information (US). PubChem Compound Summary for CID 73297272, Milademetan [Internet]. Bethesda (MD): National Library of Medicine (US); 2004 [cited 2026 May 26]. Available from: https://pubchem.ncbi.nlm.nih.gov/compound/73297272

[134] Milnerowicz S , Maszewska J , Skowera P , et al. AML under the scope: current strategies and treatment involving FLT3 inhibitors and venetoclax‐based regimens. Int J Mol Sci. 2023;24(21):15849. 37958832 10.3390/ijms242115849 PMC10647248

[135] Qureshi Z , Jamil A , Altaf F , et al. Safety, efficacy, and predictive factors of venetoclax‐based regimens in elderly acute myeloid leukemia patients: a meta‐analysis. Clin Lymphoma Myeloma Leuk. 2024;24:e835–e851. 39218712 10.1016/j.clml.2024.07.004

[136] Pan M , Wang Y , Li J , et al. Venetoclax synergizes with Wnt/β‐catenin inhibitor C‐82 in acute myeloid leukemia by increasing the degradation of Mcl‐1 protein. Cancer Cell Int. 2025;25:197. 40442688 10.1186/s12935-025-03825-8 PMC12121061

[137] Boise LH . DUB‐ling down on B‐cell malignancies. Blood. 2015;125(23):3522–3523. 26045592 10.1182/blood-2015-04-638262

[138] Desai P , Lonial S , Cashen A , et al. A phase 1 first‐in‐human study of the MCL‐1 inhibitor AZD5991 in patients with relapsed/refractory hematologic malignancies. Clin Cancer Res. 2024;30(21):4844–4855. 39167622 10.1158/1078-0432.CCR-24-0028 PMC11528199

[139] Saad J , Newman R , Khabusheva E , et al. Predictors of response and rational combinations for the novel MCL‐1 inhibitor MIK665 in acute myeloid leukemia. Mol Oncol. 2026;20(2):389–408. 40975877 10.1002/1878-0261.70130 PMC12936425

[140] Vänttinen I , Saad J , Ruokoranta T , et al. Drivers of clinical resistance to venetoclax and hypomethylating agents in acute myeloid leukemia and strategies for improving efficacy. HemaSphere. 2026;10(1):e70282. 41531664 10.1002/hem3.70282 PMC12793093

[141] Stein AS , Zhang J , Ghoda LY , et al. Phase 1 trial of navitoclax/venetoclax/decitabine combination in relapsed/refractory (R/R) acute myeloid leukemia (AML). Blood. 2024;144(suppl 1):4264.

[142] Arellano NS , Elf SE . PROTACtion against BCL‐xL in post‐MPN AML. Blood. 2025;146(3):266–267. 40674116 10.1182/blood.2025029273

[143] Wang Z , Skwarska A , Poigaialwar G , et al. Efficacy of a novel BCL‐xL degrader, DT2216, in preclinical models of JAK2‐mutated post‐MPN AML. Blood. 2025;146(3):341–355. 40163809 10.1182/blood.2024027117 PMC12333229

[144] Kadia TM , Leahy MF , Fleming S , et al. Phase Ib/II study of lisaftoclax (APG‐2575) combined with azacitidine in patients with newly diagnosed or prior venetoclax‐exposed myeloid malignancies. ASH annual meeting; 2025 Dec 7–10; Orlando, FL. Abstract 1641.

[145] Zhang J , Zhang Z , Liu J , et al. Overcoming venetoclax resistance through heme‐mediated NOXA/cyclin D1/Mcl‐1 axis with a novel artemisinin conjugate. Blood Adv. 2025;9(22):3196–3209. 10.1182/bloodadvances.2025015806 PMC12607026 40795245

[146] Zhou ZP , Tan YX , Zhao XL , et al. Venetoclax combined with three‐day multi‐frequency decitabine (DEC3‐VEN) in the treatment of adult patients with de novo acute myeloid leukemia: updated results of a phase II trial. Blood. 2025;146(suppl 1):1675.

[147] The First Affiliated Hospital of Soochow University. A prospective, randomized, open‐label study of venetoclax combined with azacitidine for consolidation therapy in adult acute myeloid leukemia [Internet]. ClinicalTrials.gov. Bethesda (MD): National Library of Medicine (US); 2026 Feb 23 [cited 2026 May 26]. Available from: https://clinicaltrials.gov/study/NCT07425782

[148] National Cancer Institute (US). Drug combo may target the unique metabolism of leukemia stem cells [Internet]. Cancer Currents Blog. 2018 Dec 14 [cited 2026 May26].Available from: https://www.cancer.gov/news‐events/cancer‐currents‐blog/2018/aml‐venetoclax‐azacitidine‐target‐leukemia‐stem‐cells

[149] He C , Xiong Y , Zeng Y , et al. HDAC3‐YY1‐RAB5A axis remodels AML‐supportive niche by modulating mitochondrial homeostasis in bone marrow stromal cells. Cell Death Dis. 2025;16(1):498. 40623992 10.1038/s41419-025-07777-9 PMC12234896

[150] Karjalainen R , Popa ML , Liu M , et al. Stroma‐derived factors stimulate JAK/STAT signaling in AML cells resulting in resistance to BCL2 inhibitor venetoclax [poster]. In: european hematology association (EHA) 22nd congress; 2017 jun 22–25; madrid, spain.

[151] Carter BZ , Mak PY , Warmoes M , et al. Inhibition of anti‐apoptotic Mcl‐1 exerts anti‐leukemia activity through modulation of leukemia‐stromal interactions and metabolic functions in AML. Blood. 2019;134(supplement 1):3727.

[152] Yu X , Munoz‐Sagredo L , Streule K , et al. CD44 loss of function sensitizes AML cells to the BCL‐2 inhibitor venetoclax by decreasing CXCL12‐driven survival cues. Blood. 2021;138(12):1067–1080. 34115113 10.1182/blood.2020006343

[153] Kuusanmäki H , Leppä AM , Pölönen P , et al. Phenotype‐based drug screening reveals association between venetoclax response and differentiation stage in acute myeloid leukemia. Haematologica. 2020;105(3):708–720. 31296572 10.3324/haematol.2018.214882 PMC7049363

[154] Zeng Z , Maiti A , Herbrich S , et al. Triple combination targeting methyltransferase, BCL‐2, and PD‐1 facilitates antileukemia responses in acute myeloid leukemia. Cancer. 2023;129(4):531–540. 36477735 10.1002/cncr.34566 PMC11884106

[155] Beijing 302 Hospital. VA Combined With PD‐1 Inhibitor for the Treatment of Relapsed and Refractory AML and High‐risk MDS [Internet]. ClinicalTrials.gov. Bethesda (MD): National Library of Medicine (US); 2024 Aug 5 [cited 2026 May 26]. Available from: https://clinicaltrials.gov/study/NCT06536959

[156] van Bruggen JAC , van der Windt GJW , Hoogendoorn M , et al. Depletion of CLL cells by venetoclax treatment reverses oxidative stress and impaired glycolysis in CD4 T cells. Blood Adv. 2022;6(14):4185–4195. 35580333 10.1182/bloodadvances.2022007034 PMC9327552

[157] Stempel J , Uy G , Dinner S , et al. Efficacy and safety of pembrolizumab added to azacitidine plus venetoclax for patients with acute myeloid leukemia: results from an investigator‐initiated, multi‐center, CTEP‐sponsored randomized, phase II trial (BLAST AML‐2) [abstract]. Blood. 2024;144(suppl 1):736.

[158] National Cancer Institute (NCI). Testing Nivolumab in Combination With Decitabine and Venetoclax in Patients With Newly Diagnosed TP53 Gene Mutated Acute Myeloid Leukemia [Internet]. ClinicalTrials.gov. Bethesda (MD): National Library of Medicine (US); 2020 Feb 20 [cited 2026 May 26]. Available from: https://clinicaltrials.gov/study/NCT04277442

[159] Dhakal P , Bates ML , Tomasson MH , et al. Acute myeloid leukemia resistant to venetoclax‐based therapy: what does the future hold? Blood Rev. 2023;59:101036. 36549969 10.1016/j.blre.2022.101036

[160] Garg R , Allen KJH , Dawicki W , et al. 225Ac‐labeled CD33‐targeting antibody reverses resistance to Bcl‐2 inhibitor venetoclax in acute myeloid leukemia models. Cancer Med. 2021;10(3):1128–1140. 33347715 10.1002/cam4.3665 PMC7897952

[161] Daver N , Montesinos P , Altman JK , et al. Pivekimab sunirine (IMGN632), a novel CD123‐targeting antibody–drug conjugate, in relapsed or refractory acute myeloid leukaemia: a phase 1/2 study. Lancet Oncol. 2024;25(3):388–399. 38423051 10.1016/S1470-2045(23)00674-5 PMC11103591

[162] Daver N , Advani A , De La Fuente Burguera A , et al. Efficacy and safety of pivekimab sunirine in combination with venetoclax plus azacitidine in unfit patients with newly diagnosed acute myeloid leukemia. Blood. 2025;146(suppl 1):651.

[163] Ong F , Kim K , Konopleva MY . Venetoclax resistance: mechanistic insights and future strategies. Can Drug Resis. 2022;5(2):380–400. 10.20517/cdr.2021.125 PMC9255248 35800373

[164] Lachowiez CA , DiNardo CD . Mutation‐ and MRD‐informed treatments for transplant‐ineligible patients. Hematology Am Soc Hematol Educ Program. 2024;2024(1):168–177. 39644009 10.1182/hematology.2024000540 PMC11665587

[165] Chen TT , Lin CC , Lo WJ , et al. Venetoclax plus azacitidine as a bridge treatment to allogeneic stem cell transplantation in unfit patients with acute myeloid leukemia. Cancers. 2024;16(6):1082. 38539418 10.3390/cancers16061082 PMC10968407

[166] Hadjis AD , McCurdy SR . Allogeneic transplantation for older adults. In: Advances in Experimental Medicine and Biology. Vol 1475. Springer; 2025:9–40. 40488822 10.1007/978-3-031-84988-6_2

[167] Walter RB , Potter V , Craddock C . Allogeneic hematopoietic cell transplantation for acute myeloid leukemia in adults over 70 years old. Blood. 2025;145(24):2847–2856. 39241195 10.1182/blood.2024024247 PMC12226756

[168] Senapati J , Kadia TM , Daver NG , et al. Therapeutic horizon of acute myeloid leukemia: success, optimism, and challenges. Cancer. 2025;131(7):e35806. 40105906 10.1002/cncr.35806

[169] Zhang J , Gu Y , Chen B . Mechanisms of drug resistance in acute myeloid leukemia. Onco Targets Ther. 2019;12: 1937–1945. 30881045 10.2147/OTT.S191621 PMC6417008

[170] Senapati J , Kantarjian HM , Bazinet A , et al. Lower intensity therapy with cladribine/low dose cytarabine/venetoclax in older patients with acute myeloid leukemia compares favorably with intensive chemotherapy among patients undergoing allogeneic stem cell transplantation. Cancer. 2024;130(19):3333–3343. 38809547 10.1002/cncr.35388 PMC12132108

[171] He H . Reduced intensity allogeneic hematopoietic stem cell transplantation in comparison to consolidation therapy based venetoclax for elderly patients with acute myeloid leukemia after first CR [Internet]. ClinicalTrials.gov. Bethesda (MD): National Library of Medicine (US); 2024 Aug 26 [cited 2026 May 26]. Available from: https://clinicaltrials.gov/study/NCT06571825

[172] Peng K , Xie J , Xu T , et al. Identification of a venetoclax‐resistance prognostic signature base on 6‐senescence genes and its clinical significance for acute myeloid leukemia. Front Oncol. 2023;13:1302356. 38098504 10.3389/fonc.2023.1302356 PMC10720639

[173] Winters AC , Minhajuddin M , Stevens BM , et al. Multi‐gene measurable residual disease assessed by digital polymerase chain reaction has clinical and biological utility in acute myeloid leukemia patients receiving venetoclax/azacitidine. Haematologica. 2024;109(6):1766–1778. 38105738 10.3324/haematol.2023.283790 PMC11141685

[174] Lu H , Wang H , Wang Q , et al. Dynamic evolution of venetoclax resistance in acute myeloid leukemia unveiled by longitudinal single‐cell RNA‐seq. Cancer Lett. 2025;628:217853. 40482912 10.1016/j.canlet.2025.217853

[175] Zhao L , Xiang X , Zhong A , et al. ASAH1‐mediated sphingolipid metabolic reprogramming in venetoclax resistance of AML: beyond the monocytic phenotypes. BMC Cancer. 2025;26:36. 41275169 10.1186/s12885-025-15272-9 PMC12781537

[176] Brown MR , Soto‐Feliciano YM . Menin: from molecular insights to clinical impact. Epigenomics. 2025;17(7):489–505. 40152985 10.1080/17501911.2025.2485019 PMC12026131

[177] Boussi L , Cai SF , Stein EM . Advances in menin inhibition in acute myeloid leukemia. Trends Cancer. 2025;11(9):889–900. 40615295 10.1016/j.trecan.2025.06.002

[178] Cluzeau T , Lemoli RM , McCloskey J , et al. Measurable residual disease in high‐risk acute myeloid leukemia. Cancers. 2022;14(5):1278. 35267586 10.3390/cancers14051278 PMC8909238

[179] Chen Y , Wang ZY , Cai RY , et al. Significant prognostic improvement in elderly patients with acute myeloid leukemia treated with hypomethylating agents and venetoclax: outcomes from a retrospective real‐world analysis. Ann Med. 2025;57(1):2512120. 40452501 10.1080/07853890.2025.2512120 PMC12131539

[180] Shimada T , Takahata A , Tanaka K , et al. Efficacy and safety of venetoclax‐based regimens versus intensive chemotherapy in older adults with newly diagnosed acute myeloid leukemia: a single‐center retrospective study. Int J Hematol. 2025;123:332–341. 41247640 10.1007/s12185-025-04111-z

